# Prevalence, distribution, and inequitable co-occurrence of mental ill-health and substance use among gender and sexuality diverse young people in Australia: epidemiological findings from a population-based cohort study

**DOI:** 10.1007/s00127-024-02714-1

**Published:** 2024-07-23

**Authors:** Sasha Bailey, Nicola Newton, Yael Perry, Cristyn Davies, Ashleigh Lin, Jennifer L. Marino, S. R. Skinner, Sophia Garlick-Bock, Ha Nguyen, Francis Mitrou, Emma Barrett

**Affiliations:** 1 The Matilda Centre for Research in Mental Health and Substance Use, Faculty of Medicine and Health, The University of Sydney, Camperdown, Australia; 2 Telethon Kids Institute, University of Western Australia, Perth, Australia; 3 Department of Adolescent Medicine, Children’s Hospital Westmead, Sydney Children’s Hospitals Network, Westmead, Australia; 4 School of Population and Global Health, University of Western Australia, Perth, Australia; 5 Children’s Hospital Westmead Clinical School, Faculty of Medicine and Health, University of Sydney, Westmead, Sydney, Australia; 6 The University of Western Australia, Perth, Australia

**Keywords:** Gender, Sexuality, Adolescents, Mental health, Substance use, Comorbidity

## Abstract

**Purpose:**

To estimate the prevalence, distribution, and co-occurrence of mental ill-health and substance use among gender and sexuality diverse young people relative to their cisgender and heterosexual peers in Australia using population-level, nationally representative data.

**Methods:**

We utilised Wave 8 (2018) data from the Longitudinal Study of Australian Children (*N* = 3037, M_age_ = 18.4) collected via an assessment protocol comprising interviews, direct observations, and assessments (on average 60 min per survey occasion). Weighted prevalence ratios and logistic regression models adjusted for demographic confounders were used to estimate the prevalence and distribution of mental ill-health (psychological distress, past 12-month self-harm thoughts and behaviours, past 12-month suicidal ideation, planning, attempt/s) and substance use outcomes (past 12-month cigarette, alcohol, and marijuana use) across gender identity (trans vs. cisgender), sexuality (gay/lesbian, bisexual, queer [those identifying with an ‘other’ sexuality identity that is not ‘gay’, ‘lesbian’, ‘bisexual’, or ‘heterosexual’] vs. heterosexual) and sexuality diversity status (sexuality diverse vs heterosexual) subgroups. Sex-stratified prevalence rates and accompanying adjusted logistic regression models were also used to assess mental ill-health and substance use disparities by sexuality diversity status. Adjusted multinominal logistic regression models were used to test disparities in co-occurring outcomes by sexuality identity) sexuality status sub-groups, and Fisher’s Exact Test of Independence for co-occurring disparities by gender identity (due to small sample size). All analyses used Wave 8 sample weights and adjusted for postcode-level clustering.

**Results:**

Among gender and sexuality diverse participants, 59 − 64% reported high or very high levels of psychological distress, 28 − 46% reported past 12-month self-harm ideation or attempts, and 26 − 46% reported past 12-month suicidal ideation, planning, or behaviour. We found significant disparities in high/very high levels of psychological distress, self-harm behaviours and suicidal behaviours among trans participants (adjusted odds ratios (aORs) ranged from 3.5 to 5.5) and sexuality diverse participants (aORs ranged from 3.5 to 3.9), compared with cisgender and heterosexual participants, respectively. Highest disparities in any past 12-month self-harm and suicidal behaviours appeared most pronounced among trans participants and queer participants compared with their cisgender, heterosexual counterparts. Minor differences by sex among sexuality diverse participants were observed for select mental ill-health outcomes. Sexuality diverse participants, and particularly sexuality diverse females, were significantly more likely to report past 12-month cigarette use and past 12-month marijuana use (adjusted odds ratio (aORs) ranging 1.4–1.6). Trans young people were at significantly elevated risk of mental ill-health in co-occurrence with cigarette and marijuana use compared with their cisgender peers (Fisher’s Exact Test of Independence *p* < 0.05 for all), whereas sexuality diverse young people were at greater risk of co-occurring mental ill-health and cigarette co-use and marijuana co-use, compared with their non-sexuality diverse peers (adjusted multinomial odds ratios (aMORs) ranging 2.2-6.0).

**Conclusion:**

Mental ill-health, substance use, and their co-occurrence disproportionately affects gender and sexuality diverse young people in Australia. Further research should study the longitudinal development of these disparities through adolescence, with close attention to the social, embodied contexts of substance use among LGBTQ + young people with the view to building LGBTQ + affirming models of harm reduction.

**Supplementary Information:**

The online version contains supplementary material available at 10.1007/s00127-024-02714-1.

## Introduction

While many gender and sexuality diverse (LGBTQ+) young people live healthy, fulfilled lives, higher rates of mental ill-health among LGBTQ + young people relative to their cisgender and heterosexual peers have been documented in Australia and worldwide [1–5]. For example, the Writing Themselves In 4 survey of 6,418 LGBTQ + young people aged 14–21 in Australia found over 80% of participants reported high or very high levels of psychological distress, over 40% reported past 12-month self-harm, and up to 77% reported past 12-month suicidal ideation, thoughts or attempts [1]. However, these estimates have significant heterogeneity across LGBTQ + subgroups. The Trans Pathways survey of 859 trans, non-binary, and gender diverse (henceforth respectfully referred to with the umbrella term, ‘trans’) young people in Australia found approximately 75% of trans young people reported a depression diagnosis; 80% reported having ever self-harmed, and 48% reported attempting suicide at some stage in their lifetime [2, 6]. Similar rates of mental ill-health among LGBTQ + young people have been reported in other jurisdictions worldwide, including a disproportionately higher burden among trans young people [5, 7]. A significant limitation of many of these prevalence studies, however, is the lack of population-level representativeness due to the use of convenience sampling methods, inadequate sample size/statistical power, the conflation of gender and sexuality, or lack of a cisgender and/or heterosexual comparison group. Accurate, population-based research is critical for understanding and preventing mental ill-health and related costs across the lifespan.

LGBTQ + young people are also at increased risk of substance use relative to their cisgender and heterosexual peers [8]. Research shows that substance use is more prevalent [9] and initiated at earlier ages [10] among LGBTQ + young people relative to cisgender and heterosexual young people, particularly cigarette, alcohol and marijuana use [11–14]. This warrants significant public health attention because mental ill-health and substance use typically emerge and co-occur during adolescence and share common, interacting and compounding risk factors [15–17]. It is important to note that substance use in and of itself does not equate to substance use-related harms. Accordingly, past studies have found that many LGBTQ + young people engage in substance use for altruistic, social, and community-seeking motivations [18, 19]. Notwithstanding this, compared with individual occurrences, co-occurring mental ill-health and substance use bears significantly higher burden and morbidity and requires different strategies for prevention, early intervention and treatment [20, 21]. Moreover, the risk factors which predispose co-occurrence may also create other vulnerabilities, including sexual risk-taking and adolescent behaviour problems [22]. Despite the significant burden of mental ill-health and substance use among LGBTQ + young people, current literature is siloed and does not consider the prevalence and distribution of co-occurring mental ill-health and substance use among LGBTQ + young people [23]. Scarce available literature regarding co-occurring mental ill-health and substance use among LGBTQ + people focuses predominantly on sexuality diverse groups [24] and LGBTQ + adults [25–27]. A recent study using national cohort data of LGBTQ + young adults in the United States was conducted to estimate the prevalence of co-occurring depressive symptoms and probable substance use disorders; however, this study was limited to LGBTQ + people aged 20–35 and did not stratify results by participants’ ages [28].

Further research into co-occurring mental ill-health and substance use should estimate prevalence among LGBTQ + young people of high school-leaving age (i.e., 18 years), typically associated with the onset and peak of substance use-related harms in mainstream populations [29]. Whereas previous research has utilised disorder-level measures of co-occurring mental ill-health and substance use among LGBTQ + people, there is scarce literature that utilises non-specific or transdiagnostic psychiatric screening tools and self-report measures of frequency of substance use [30]. Screening and frequency-based tools more comprehensively capture substance use harms among this population because substance use during adolescence generally is associated with significant neurodevelopment harms and is a significant risk factor for developing and onset substance use disorders during adulthood [21]. Lastly, current literature on co-occurring adolescent mental health and substance use disorders does not include self-harm and suicidal behaviours in the context of mental ill-health [24, 28]. Self-harm and suicidal behaviours disproportionately affect LGBTQ + young people [31, 32] and are significantly associated with substance use [33]. To our knowledge, little to no research has been conducted to investigate the potential co-occurrence of self-harm and suicidal behaviours with substance use among LGBTQ + young people.

Over the past two decades, a significant body of high-quality, LGBTQ + communities-engaged research has emerged, providing vital insights into the burden, magnitude and nature of mental ill-health and substance use among LGBTQ + young people in Australia, forming a vital evidence basis for real-life public health policy and practice change for LGBTQ + communities. These include the Writing Themselves In [1], Trans Pathways [2], Growing Up Queer [34], The First Australian National Trans Mental Health [35], and the From Blues to Rainbows [36] studies. A significant limitation of many of these prevalence studies, however, is the lack of population-level representativeness due to the use of convenience sampling methods, inadequate sample size/statistical power, the conflation of gender and sexuality, or lack of a cisgender and/or heterosexual comparison group. Accurate, population-based research is critical for understanding and preventing mental ill-health and related costs across the lifespan.

The present study aimed to: (1) estimate the prevalence of mental ill-health and substance use among LGBTQ + young people; and (2) compare differences in mental ill-health, substance use, and co-occurring mental ill-health and substance use outcomes between trans vs. cisgender and sexuality diverse vs. heterosexual participants in a population-level, nationally representative sample in Australia.

## Methods

### Study design and participants

The Longitudinal Study of Australian Children (LSAC) is a cross-sequential study comprising two 12-month age cohorts (B cohort, infants aged 0–1 years, and K cohort, children aged 4–5 years old when the study began). This study used data from Wave 8 of the K cohort, who were born between March 1999 and February 2000 and were followed up in 2018 when they were aged 17–19. The Wave 8 response rate was approximately 77.3%, and Wave 1 retention rate was approximately 61.1% [37]. The LSAC sample was selected from the Medicare Australia enrolments database, Australia’s most comprehensive population database, particularly of young children [38]. A two-stage clustered design was employed wherein 311 geographic postcodes were randomly selected, following which, children were subsequently randomly selected within each postcode [38]. Stratification was utilised to ensure that numbers of children selected were roughly proportionate to the total number of children within each Australian state/territory, capital city districts and broader regional surrounding areas [38]. This method of accounting for the number of children in each postcode meant that potentially participants across Australia had an approximately equal chance of selection (approximately one in 25) [38]. Full detail regarding using a non-probability-based selection of participations via geographically representative postcode sampling with a homogenous probability of selection is published elsewhere [38]. LSAC methodology and survey content have received ethical review and approval by the Australian Institute of Family Studies Ethics Committee.

### Measures

#### Sexuality identity (individual-level sexuality diversity)

Participants were asked, “Which of the following categories best describes how you think of yourself” with options including ‘Heterosexual or straight’, ‘Gay or lesbian’, ‘Bisexual’, ‘Other’, and ‘Don’t know’.

#### Sexuality diversity (group-level sexuality diversity)

A ‘Sexuality Diversity’ variable was computed where Sexuality Diversity included those who indicated that their sexuality was ‘Gay or Lesbian’, ‘Bisexual’, ‘Other’, or ‘Don’t know’. Additionally, a sexual attraction item was asked of participants: ‘Which of these statements best describes your sexual feelings at this time in your life?’ with possible responses including ‘Only attracted to females’, ‘Mostly attracted to females’, ‘I’m equally attracted to females and males’, ‘Mostly attracted to males’, ‘Only attracted to males’, ‘Never felt attracted to anybody at all’, and ‘Unsure’. Male participants who responded that they were only, mostly, or equally attracted to males, female participants who responded that they were only, mostly, or equally attracted to females, and participants who responded that they had never felt attracted to anybody or were unsure about their sexual feelings, were coded as ‘Sexuality Diverse’. A third item was used for this Sexuality Diversity variable assessing the sex of participants’ past sexual partners: ‘Which of the following options best describes who you have had sex with during the last 12 months?’ Responses were ‘Only with males’, ‘Only with females’, and ‘With both males and females’. Male participants who reported that they had had sex only with males, female participants who reported that they had had sex only with females, and participants who responded that they had had sex with both males and females, were also coded as ‘Sexuality Diverse’. Those who did not meet these criteria were coded as ‘Non-Sexuality Diverse’.

#### Gender identity

Participants were asked about their Sex (Male or Female) and Gender Identity (Male; Female; Transgender, male to female; Transgender, female to male; Genderqueer; and Other). Trans people were participants who explicitly identified as transgender, and those who indicated Gender Identity different from Sex. Participants with concordant Gender Identity and Sex responses were classified as Cisgender.

#### Mental ill-health

Past Australia-specific research examining distributions of mental ill-health among LGBTQA + young people has concentrated on psychological distress [1] and experiences of self-harm [1, 6] and suicidality [1, 6] thus the researchers sought to analyse those identical or similar concordant measures of psychological distress, self-harm, and suicidality within LSAC to enable cross-cohort comparisons.

##### Psychological distress

The 10-item Kessler Psychological Distress Scale (K10) was administered to participants to capture past four-week non-specific psychological distress [39]. A scale score is calculated by summing scores for each item with higher scores indicating greater psychological distress. Previous research has shown that the K10 measure possesses sound factor structure and predictive validity in Australian child and adolescent populations [40]. As defined by the Australian Bureau of Statistics, scores *≥* 22 indicate high or very high levels of psychological distress [39, 41].

##### Self-harm thoughts and behaviour

Participants indicated ‘yes’ or ‘no’ to whether, in the past 12 months they had ‘thought about hurting (themselves) on purpose in any way?’ or ‘hurt (themselves) on purpose in any way?’.

##### Suicidal thoughts and behaviours

Participants were asked (yes/no) whether, during the past 12 months, they had ‘ever seriously consider(ed) attempting suicide?’ or ‘made a plan about how (they) would attempt suicide?’ Participants were also asked, ‘During the past 12 months, how many times did you actually attempt suicide? (0 times, 1 time, 2 or 3 times, 4 or 5 times, 6 or more times). Suicide attempts were categorised and dichotomised where *≥* 1 indicated a past 12-month suicide attempt, and 0 indicated no past 12-month suicide attempt.

#### Substance use

##### Past 12-month cigarette, alcohol, and marijuana use

Six separate items assessed participants’ ever use and past 12-month use of cigarettes, alcohol, and marijuana (yes/no items). For each substance use outcome, participants were coded as indicating past 12-month use if they reported ever use *and* past 12-month use for a given substance. Participants were coded as not reporting past 12-month use if they indicated they had never used a given substance *or* had not used that substance use in the past 12-months. Due to low sample sizes, the researchers were unable to analyse responses to other drugs, such as hallucinogens, methamphetamine, and opioids. Similarly, low cell counts prevented the researchers from conducting gender and sexuality-specific sub-group analyses using past 4-week substance use, past 7-day substance, recent participation in hazardous drinking, and current smoking status. Notwithstanding this, much of the existing literature related to substance use among LGBTQ + young people specifically (and not adults) in Australia focuses on the burden of cigarette use and alcohol consumption [1, 6] and less about use of other ‘illicit drugs’ [42] hence the researchers were still able to make meaningful cross-cohort comparisons.

### Co-occurring mental ill-health and substance use

Utilising the same approach implemented in a previous study of co-occurring depressive symptoms and probable substance use disorders among LGBTQ + people [28], we created nine new variables for co-occurring mental ill-health and substance use:

1. *High/very high levels of psychological distress and past 12-month cigarette use*: (a) no past 4-week high or very high levels of psychological distress, no past 12-month cigarette use, (b) past 4-week high or very high levels of psychological distress only (c) past 12-month cigarette use only, (d) both past 4-week high or very high levels of psychological distress and past 12-month cigarette use.
2. *High/very high levels of psychological distress and past 12-month alcohol use*: (a) no past 4-week high or very high levels of psychological distress, no past 12-month alcohol use, (b) past 4-week high or very high levels of psychological distress only, (c) past 12-month alcohol use only, (d) both past 4-week high or very high levels of psychological distress and past 12-month alcohol use.
3. *High/very high levels of psychological distress and past 12-month marijuana use*: (a) no past 4-week high or very high levels of psychological distress, no past 12-month marijuana use, (b) past 4-week high or very high levels of psychological distress only, (c) past 12-month marijuana use only, (d) both past 4-week high or very high levels of psychological distress and past 12-month marijuana use.
4. *Past 12-month self-harm thoughts/behaviours and past 12-month cigarette use*: (a) no past 12-month self-harm ideation or attempts, no past 12-month cigarette use, (b) past 12-month self-harm ideation or attempts only, (c) past 12-month cigarette use only, (d) both past 12-month self-harm ideation or attempts and past 12-month cigarette use.
5. *Past 12-month self-harm thoughts/behaviours and past 12-month alcohol use*: (a) no past 12-month self-harm ideation or attempts, no past 12-month alcohol use, (b) past 12-month self-harm ideation or attempts only, (c) past 12-month alcohol use only, (d) both past 12-month self-harm ideation or attempts and past 12-month alcohol use.
6. *Past 12-month self-harm thoughts/behaviours and past 12-month marijuana use*: (a) no past 12-month self-harm ideation or attempts, no past 12-month marijuana use, (b) past 12-month self-harm ideation or attempts only, (c) past 12-month marijuana use only, (d) both past 12-month self-harm ideation or attempts and past 12-month marijuana use.
7. *Past 12-month suicidal thoughts/behaviours and past 12-month cigarette use*: (a) no past 12-month suicidal ideation, planning or attempts, no past 12-month cigarette use, (b) past 12-month suicidal ideation, planning or attempts only, (c) past 12-month cigarette use only, (d) both past 12-month suicidal ideation, planning or attempts and past 12-month cigarette use.
8. *Past 12-month suicidal thoughts/behaviours and past 12-month alcohol use*: (a) no past 12-month suicidal ideation, planning or attempts, no past 12-month alcohol use, (b) past 12-month suicidal ideation, planning or attempts only, (c) past 12-month alcohol use only, (d) both past 12-month suicidal ideation, planning or attempts and past 12-month alcohol use.
9. *Past 12-month suicidal thoughts/behaviours and past 12-month marijuana use*: (a) no past 12-month suicidal ideation, planning or attempts, no past 12-month marijuana use, (b) past 12-month suicidal ideation, planning or attempts only, (c) past 12-month marijuana use only, (d) both past 12-month suicidal ideation, planning or attempts and past 12-month marijuana use.

### Data analyses

For descriptive statistics, categorical variables were summarised with counts and proportions and continuous variables, with means and standard deviations. Generalised linear models were used to adjust for postcode-level clustering. Multivariate logistic regression models controlling for age, sex, socio-economic status, and region of residence were used to calculate adjusted odds ratios to compare differences in prevalence of mental ill-health (high/very high level of psychological distress, past 12-month self-harm ideation and attempts, and past 12-month suicidal ideation, planning and attempts) and substance use (past 12-month cigarette use, alcohol use, and marijuana use) between: heterosexual participants and gay/lesbian, bisexual, and other-sexuality participants (categorised separately); non-sexuality-diverse and sexuality diverse participants; and trans and cisgender participants. For these logistic regression models, referent groups comprised heterosexual, non-sexuality-diverse, and cisgender participants, respectively. Sex-stratified weighted prevalence statistics and multivariate logistic regression models controlling for age, sex, socio-economic status, and region of residence were also computed to describe and test sex-varying differences in mental ill-health and substance use between sexuality diverse vs. non-sexuality diverse participants. Multinomial logistic regression estimates controlling for age, sex, socio-economic status, and region of residence, were conducted to test for associations between sexuality diversity and co-occurring mental ill-health and substance use outcomes. Utilising multinomial logistic regression to analyse dependent variables with several, unordered categories instead of collapsing dependent variables into two mutually exclusive groups to utilise binary logistic regression, maintains higher statistical power, does not require the satisfaction of assumptions regarding normality, linearity, or homoscedasticity, and provides easy interpretability. Furthermore, multinominal logistic regression analysis assumes non-perfect separation, which is particularly salient to the present study given that many young people who do not have co-occurring mental ill-health and substance use may still have mental ill-health or substance use, rather than neither. Due to the small sample size of trans participants, Fisher’s Exact Tests of Independence were performed to detect associations between participants’ gender identity and co-occurring mental ill-health and substance use outcomes.

Unadjusted estimates for models detailed above are presented in Supplementary Material B. Age was selected as covariates given the exponential rise of mental ill-health and substance use during adolescence; just under half (48.4%) of all mental disorders onset before age 18 [29]. Sex was also identified as a covariate given past research has shown that the co-occurrence of mental ill-health and substance use among LGBTQ + young people differs by sex presumed at birth [24]. Lastly, socio-economic status and region of residence were included as model covariates given their interaction and association with mental ill-health and substance use among young people [43].

There was < 13% missing data affecting all mental ill-health outcomes assessed in this study (psychological distress, self-harm thoughts and behaviours, suicide thoughts and behaviours). However further inspection stratifying these measures by gender and sexuality found that < 1% of participants reporting missing data for any of these mental ill-health outcomes also reported gender and sexuality data. Hence, to handle this missing data, complete case analysis was used excluding participants who did not record complete data on gender and sexuality as well as mental ill-health outcomes. To further mitigate impacts of missing data, complete case analysis was also used for past 12-month substance use outcomes wherein eligible participants included in the substance use analyses were required to record valid responses (yes or no) to both ‘ever use’ *and* ‘past 12-month use’ substance use items. Complete case analysis was also used for multinomial models testing co-occurring mental ill-health and substance use disparities by sexuality diversity, and Fisher’s Exact Tests of Independence for disparities by gender.

Given the exploratory nature of this epidemiological work, a statistically significant threshold of *p* < 0.05 was used for all models. All analyses utilised Wave 8 composite sample weights to correct for participant attrition over waves and to realign samples with the original probability sample design of the study when K-cohort participants were 4–5 years old. All generalised linear models, including logistic regression models, adjusted for postcode-level clustering. All statistical analyses were conducted using statistical software, RStudio (Version 4.2.2). The present study was prepared in accordance with the guidelines outlined in the Strengthening the reporting of observational studies in epidemiology (STROBE) checklist for cross-sectional studies [44]. An annotated copy of this checklist is provided in Supplementary Material Appendix A.

## Results

Among the 3,037 K-cohort young people participating in Wave 8 of LSAC data collection (M_age_ = 18.4, SD_age_ = 0.5), 2,261 (87.6%) identified as heterosexual/straight, 56 (2.2%) gay/lesbian, 225 (8.7%) bisexual, and 39 (1.5%) ‘other’ sexuality (henceforth referred to respectfully as “those with other sexualities”). At an overall group-level categorisation of sexuality diversity, 402 participants (14.3%) were sexuality diverse and 2,211 participants (85.6%) were non-sexuality diverse. Of the total sample, there were 36 (1.4%) trans participants and 2,619 (98.6%) cisgender participants. More detail regarding the demographic characteristics of these LGBTQ + groups and their heterosexual (identity-level), non-sexuality diverse (group-level), and cisgender comparators is available in Table 1.

**Table 1:** Demographic characteristics of young people by gender, sexuality identity, and sexuality diversity

	Heterosexual	Gay/Lesbian	Bisexual	Other	Non-Sexuality Diverse	Sexuality Diverse	Cis	Trans
All (N)	2261	56	225	39	2211	402	2619	36
Age – M (SD)	18.4 (0.5)	18.5 (0.5)	18.4 (0.5)	18.3 (0.47)	18.4 (0.5)	18.3 (0.5)	18.4 (0.5)	18.4 (0.5)
Binary sex recorded at birth								
Female – N (%)	1026 (45.9)	25 (39.9)	172 (71.8)	24 (74.4)	998 (45.8)	268 (63.4)	1278 (49.0)	22 (56.9)
Male – N (%)	1210 (54.1)	37 (60.1)	67 (28.2)	*	1182 (54.2)	155 (36.6)	1333 (51.0)	17 (43.1)
Indigenous status								
Aboriginal and/or Torres Strait Islander – N (%)	68 (3.0)	*	11 (4.6)	*	62 (2.9)	27 (6.3)	84 (3.2)	*
*Australian state of residence – N*								
NSW	741	27	84	11	725	151	870	17
VIC	540	*	44	*	523	90	621	*
QLD	470	12	56	*	456	94	547	*
SA	154	*	17	*	150	26	176	*
WA	219	*	22	*	216	36	256	*
TAS	59	*	*	*	59	15	75	*
NT	*	*	*	*	*	*	10	*
ACT	44	*	*	*	44	*	56	*
*Region of residence N (%)*								
Non-metropolitan	777 (34.8)	24 (38.7)	78 (32.8)	*	760 (34.9)	141 (33.3)	902 (34.6)	18 (47.5)
Metropolitan	1454 (65.2)	38 (61.3)	160 (67.2)	23 (71.3)	1416 (65.1)	282 (66.7)	1704 (65.4)	20 (52.5)
Index of Relative Socio-economic Disadvantage Scores – M (SD)	1019 (65.9)	1003 (81.5)	1010 (68.8)	1031 (58.6)	1012 (69.8)	1019 (66.1)	1018 (66.9)	1017 (70.7)

*Cell counts *≤* 10 are censored to prevent re-identification of participants as required by the Australian Institute of Family Studies

### Prevalence and distribution of mental ill-health

Table 2 reports prevalence of mental ill-health, substance use and co-occurring outcomes among LGBTQ + young people in our sample. Among trans young people in this sample, 64% experienced high or very high levels of psychological distress, 35% reported past 12-month self-harm thoughts/behaviours, and 46% reported past 12-month suicidal thoughts/behaviours. Trans participants were at significantly increased odds of high or very high levels of psychological distress, self-harm thoughts/behaviours, and suicidal thoughts/behaviours compared with their cisgender peers (adjusted odds ratios (aORs) 3.5–5.5) regardless of age, sex, relative socio-economic status, and region of residence.

**Table 2:** Prevalence and distribution of psychological distress, self-harm, and suicidality among LGBTQ + young people in the longitudinal study of Australian children

	Trans vs. Cis			Heterosexual vs. Gay/Lesbian / Bisexual / Other							Non-Sexuality diverse vs. Sexuality Diverse			Female Non-Sexuality Diverse vs. Female Sexuality Diverse			Male Non-Sexuality Diverse vs. Male Sexuality Diverse		
	Cis (ref.)	Trans		Heterosexual (ref.)	Gay/Lesbian		Bisexual		Other		Non-Sexuality Diverse (ref.)	Sexuality Diverse		Female Non-Sexuality Diverse (ref.)	Female Sexuality Diverse		Male Non-Sexuality Diverse (ref.)	Male Sexuality Diverse	
	N %	N %	aOR 95% CI *p*	N %	N %	aOR 95% CI *p*	N %	aOR 95% CI *p*	N %	aOR 95% CI *p*	N %	N %	aOR 95% CI *p*	N %	N %	aOR 95% CI *p*	N %	N %	aOR 95% CI *p*
High or very high levels of psychological distress	871 33.4	25 63.6	3.5 1.5, 8.5 0.005	648 29.0	42 68.3	5.7 2.7, 12.0 < 0.001	155 64.8	3.9 2.7, 12.0 *< 0.001*	19 59.3	3.0 1.4, 6.4 *0.004*	623 28.6	248 58.7	3.7 2.8, 4.8 *< 0.001*	364 36.6	157 61.3	3.2 2.2, 4.5 *< 0.001*	258 21.9	73 49.8	4.1 2.6, 6.4 *< 0.001*
Any past 12-month self-harm thoughts/behaviour	382 14.7	18 45.5	4.7 2.1, 10.4 *< 0.001*	262 11.7	44 28.4	3.0 1.6, 5.8 *< 0.001*	91 38.5	4.3 2.9, 6.3 *< 0.001*	14 44.0	5.4 2.7, 10.9 *< 0.001*	247 11.4	139 33.3	4.0 3.0, 5.5 *< 0.001*	132 13.3	88 34.8	3.8 2.5, 5.8 *< 0.001*	113 9.6	39 26.5	4.0 2.4, 6.7 *< 0.001*
Past 12 months self-harm thoughts	307 11.8	14 35.2	4.0 1.7, 9.1 *0.001*	210 9.4	16 26.2	3.3 1.6, 6.9 *< 0.001*	72 30.2	3.8 2.5, 5.8 *< 0.001*	13 41.6	6.7 3.3, 13.5 *< 0.001*	197 9.0	117 27.8	4.0 2.8, 5.5 *< 0.001*	101 10.1	76 29.8	4.1 2.6, 6.3 *< 0.001*	94 7.9	32 22.1	3.7 2.1, 6.3 *< 0.001*
Past 12 months self-harm behaviour	234 9.0	14 35.4	5.5 2.5, 12.2 *< 0.001*	159 7.1	14 23.5	4.1 2.0, 8.4 *< 0.001*	55 23.3	3.6 2.3, 5.6 *< 0.001*	*	3.8 1.7, 8.6 *0.001*	146 6.7	90 21.5	4.0 2.8, 5.7 *< 0.001*	80 8.1	54 21.3	3.4 2.1, 5.5 *< 0.001*	65 5.5	26 17.4	4.4 2.3, 8.4 *< 0.001*
Any past 12-month suicidal thoughts/behaviour	396 15.2	18 46.3	4.7 2.2, 10.1 *< 0.001*	280 12.5	18 29.1	2.8 1.4, 5.5 *0.003*	91 38.3	4.1 2.8, 5.9 *< 0.001*	14 44.3	5.4 2.8, 10.7 *< 0.001*	267 12.2	140 33.2	3.7 2.7, 5.0 *< 0.001*	130 13.1	89 35.1	3.9 *2.6, 5.8* *< 0.001*	134 11.4	39 26.3	3.3 2.0, 5.5 *< 0.001*
Past 12 months suicidal thoughts	307 11.8	14 35.2	4.0 1.7, 9.1 *0.001*	210 9.4	16 26.2	3.3 1.6, 6.9 *< 0.001*	72 30.2	3.8 2.5, 5.8 *< 0.001*	13 41.6	6.7 3.3, 13.5 *< 0.001*	197 9.0	117 27.8	4.0 2.8, 5.5 *< 0.001*	101 10.1	76 29.8	4.1 *2.7, 6.3* *< 0.001*	94 7.9	32 22.1	3.8 2.2, 6.5 *< 0.001*
Past 12 months suicidal planning	234 9.0	13 34.1	5.2 2.4, 11.1 *< 0.001*	168 7.5	12 20.2	2.9 1.3, 6.6 *0.009*	50 21.1	3.2 2.1, 5.0 *< 0.001*	*	3.8 1.6, 8.8 *0.002*	157 7.2	84 19.9	3.3 2.4, 4.7 *< 0.001*	70 7.1	48 18.9	3.1 1.9, 5.0 *< 0.001*	86 7.3	26 17.7	3.1 1.7, 5.6 *< 0.001*
Past 12 months suicide attempt	151 5.8	*	3.0 1.1, 8.1 *0.03*	103 4.6	*	3.8 1.5, 9.8 *0.005*	36 14.9	3.4 2.0, 5.7 *< 0.001*	*	2.1 0.5, 9.2 *0.3*	94 4.3	58 13.6	3.6 2.3, 5.7 *< 0.001*	49 4.9	32 12.7	3.0 1.6, 5.6 *< 0.001*	46 3.8	20 13.5	4.3 2.1, 8.9 *< 0.001*
Past 12 months alcohol use	29 78.0	2100 (87.2)	0.50.2, 1.4 *0.2*	1791 (86.8)	51 (87.5)	1.2 0.3, 3.5 *0.8*	212 (93.2)	2.2 1.1, 4.25 *0.02*	17 (64.0)	0.3 0.1, 0.6 *< 0.001*	1760 (87.4)	310 (85.9)	0.90.6, 1.4 *0.6*	822 (87.5)	191 (87.7)	1.1 0.6, 1.9 *1.2*	937 (87.3)	101 (82.2)	0.7 0.4, 1.3 *0.3*
Past 12 months cigarette use	18 (50.8)	875 (36.9)	1.7 0.8, 3.9 *0.2*	733 (36.1)	28 (48.9)	1.6 0.9, 3.1 *0.1*	104 (46.5)	1.6 1.1, 2.3 *0.008*	*	0.5 0.2, 1.2 *0.1*	715 (36.2)	156 (43.1)	1.4 1.0, 1.8 *0.02*	298 (32.6)	92 (42.9)	1.61.1, 2.2 *0.008*	416 (39.1)	54 (42.3)	1.1 0.7, 1.7 *0.7*
Past 12 months marijuana use	*	706 (22.9)	0.70.3, 1.9 *0.5*	580 (28.3)	20 (36.0)	1.5 0.8, 2.9 *0.2*	96 (42.4)	2.2 1.5, 3.1 *< 0.001*	*	0.4 0.1, 1.2 *0.1*	568 (28.4)	132 (36.4)	1.6 1.2, 2.2 *0.001*	219 (23.6)	79 (36.2)	1.9 1.3, 2.7 *< 0.001*	348 (32.5)	46 (36.9)	1.3 0.8, 2.1 *0.3*

*Cell counts *≤* 10 are censored to prevent re-identification of participants as required by the Australian Institute of Family Studies

Among gay/lesbian participants, 68% reported high or very high levels of psychological distress, 28% reported past 12-month self-harm thoughts/behaviours, and 29% reported past 12-month suicidal thoughts/behaviours. Among bisexual participants, 65% reported high or very high levels of psychological distress, 39% reported past 12-month self-harm thoughts/behaviours, and 38% reported past 12-month suicidal thoughts/behaviours. Among people with other sexualities, 59% reported high or very high levels of psychological distress, 44% reported past 12-month self-harm thoughts/behaviours, and 44% reported past 12-month suicidal thoughts/behaviours. Irrespective of differences related to age, sex, socio-economic status, and region of residence, compared with their heterosexual peers, gay/lesbian and bisexual people, and people with other sexualities were at significantly increased odds of experiencing high or very high levels of psychological distress, self-harm thoughts/behaviours, and suicidal thoughts/behaviours (aORs ranged between 2.9 and 5.3, 4.2–4.7, and 3.6–5.5, respectively).

At a group level, among sexuality diverse participants 59% reported high or very high levels of psychological distress, 33% reported past 12-month self-harm thoughts/behaviours, and 33% reported past 12-month suicidal thoughts/behaviours. Among sexuality diverse young females, 61% reported high or very high levels of psychological distress, 35% reported past 12-month self-harm thoughts/behaviours, and 35% reported past 12-month suicidal thoughts/behaviours. Among sexuality diverse young males, 50% reported high or very high levels of psychological distress, 27% reported past 12-month self-harm thoughts/behaviours, and 26% reported past 12-month suicidal thoughts/behaviours. Sexuality diverse people had significantly increased odds of experiencing all mental ill-health indicators, compared with their non-sexuality diverse peers (aORs ranged from 3.5 to 3.9) after controlling for age, sex, socio-economic status, and region of residence. Compared with non-sexuality diverse young males and females, sexuality diverse males and females were at increased odds of experiencing high or very high levels of psychological distress, self-harm thoughts/behaviours, and suicidal thoughts/behaviours (aORs ranged between 2.8 and 3.5 and 2.7–3.6, respectively), after adjusting for differences in age, sex, socio-economic status, and region of residence. Sensitivity analyses comprising unadjusted models testing crude differences in mental ill-health by gender, sexuality, and sexuality diversity are presented in Supplementary Material B.

### Prevalence and distribution of substance use

As shown in Table 1, across all groups, alcohol was the most commonly used substance, tobacco the second most commonly used, and marijuana the least commonly used. Substance use prevalence varied between 23% and 78% among trans participants; 36 − 88% among gay/lesbian participants; 43 − 93% among bisexual participants; and 12 − 64% among participants with other sexualities, depending on substance type. At a group level, substance use prevalence varied between 36 − 86% among sexuality diverse participants; 36 − 88% among sexuality diverse females; and 37 − 82% among sexuality diverse men, depending on substance use type. As shown in Table 2, no statistically significant risk differences of any substance use outcomes were observed between trans vs. cisgender participants, gay/lesbian vs. heterosexual participants, and people with other sexualities vs. heterosexual participants, after accounting for age, sex, socio-economic status, and region of residence.

Bisexual people were at increased odds of reporting all past 12-month substance use outcomes assessed in this study, namely alcohol use, cigarette use, and marijuana use, regardless of differences in age, sex, socio-economic status, and region of residence (aORs ranging 1.6–2.2). Young people with other sexualities reported significantly decreased odds of past 12-month alcohol use, after controlling for confounders (aOR = 0.3).

At a group-level, relative to non-sexuality diverse participants, sexuality diverse participants were at significantly increased odds of reporting past 12-month cigarette use and past 12-month marijuana use after controlling for age, sex, socio-economic status, and region of residence (aORs ranging 1.4–1.6). Relative to female non-sexuality diverse participants, female sexuality diverse participants also reported significantly increased odds of past 12-month cigarette use and past 12-month marijuana use, regardless of differences related to age, sex, socio-economic status, and region of residence (aORs ranging 1.6–1.9).

No significant disparities in any past 12-month substance use outcomes were detected among trans participants relative to their cisgender peers, gay/lesbian participants relative to their heterosexual peers, and sexuality diverse males compared to non-sexuality diverse males, after controlling for differences in age, sex, socio-economic status, and region of residence.

Sensitivity analyses comprising unadjusted models testing crude associations differences in substance use by gender, sexuality, and sexuality diversity are presented in Supplementary Material B.

### Gender and sexuality differences in co-occurring mental ill-health and substance use

Overall, relative to non-sexuality diverse peers, sexuality diverse young people were at increased odds of reporting all nine co-occurring mental ill-health and substance use outcomes assessed in this study, regardless of age, sex, socio-economic status, and region of residence (Table 3). Namely, sexuality diverse young people were at increased odds of co-occurring high or very high levels of psychological distress and substance use, particularly cigarette use (adjusted multinomial odds ratio (aMOR) = 4.7, 95% CI: 3.4–6.5, *p* < 0.001); alcohol use (aMOR = 2.2, 95% CI: 1.7–2.8, *p* < 0.001); and marijuana use (aMOR = 5.3, 95% CI: 3.8–7.4, *p* < 0.001), after controlling for age, sex, socio-economic status, and region of residence. Sexuality diverse young people were also at increased odds of engaging in self-harm thoughts/behaviours in co-occurrence with cigarette use (aMOR = 5.6, 95% CI: 3.9-8.0, *p* < 0.001); alcohol use (2.8, 95% CI: 1.9–4.1, *p* < 0.001); and marijuana use (aMOR = 6.0, 95% CI: 4.1–8.8, *p* < 0.001), after adjustment for age, sex, socio-economic status, and region of residence. Sexuality diverse young people were also at increased odds of engaging in suicidal thoughts/behaviours in co-occurrence with cigarette use (aMOR = 5.3, 95% CI: 3.7–7.6, *p* < 0.001); alcohol use (aMOR = 2.8, 95% CI: 1.9-4.0, *p* < 0.001); and marijuana use (aMOR = 5.4, 95% CI: 3.7–7.9, *p* < 0.001), regardless of age, sex, socio-economic status, and region of residence.

**Table 3:** Multivariate multinominal logistic regression models estimating associations between sexuality diversity with outcomes of co-occurring mental ill-health and substance use among sexuality diverse young people

	Sexual orientation		
	Non-Sexuality Diverse	Sexuality Diverse	
	aMOR^1^	aMOR (95% CI)^2^	*p*-value
Outcome 1: High or very high levels of psychological distress (K10^3^) and Recent Cigarette Use (CU^4^)			
No K10, No CU	1.0	-	-
K10 Only	1.0	3.6 (2.6, 4.9)	< 0.001
CU Only	1.0	1.1 (0.8, 1.6)	0.5
K10 and CU	1.0	4.7 (3.4, 6.5)	< 0.001
Outcome 2: High or very high levels of psychological distress (K10) and Recent Alcohol Use (AU^5^)			
No K10, No AU	1.0	-	-
K10 Only	1.0	1.7 (1.3, 2.3)	< 0.001
AU Only	1.0	0.5 (0.4, 0.6)	< 0.001
K10 and AU	1.0	2.2 (1.7, 2.8)	< 0.001
Outcome 3: High or very high levels of psychological distress (K10) and Recent Marijuana Use (MU^6^)			
No K10, No MU	1.0	-	-
K10 Only	1.0	3.7 (2.8, 5.0)	< 0.001
MU Only	1.0	1.4 (0.9, 2.1)	0.1
K10 and MU	1.0	5.3 (3.8, 7.4)	< 0.001
Outcome 4: Recent Self-harm Behaviours (SH^7^) and Recent Cigarette Use (CU)			
No SH, No CU	1.0	-	-
SH Only	1.0	3.3 (2.3, 4.9)	< 0.001
CU Only	1.0	1.1 (0.9, 1.5)	0.4
SH and CU	1.0	5.6 (3.9, 8.0)	< 0.001
Outcome 5: Recent Self-harm Behaviours (SH) and Recent Alcohol Use (AU)			
No SH, No AU	1.0	-	-
SH Only	1.0	1.4 (1.3, 1.6)	< 0.001
AU Only	1.0	0.6 (0.4, 0.8)	0.003
SH and AU	1.0	2.8 (1.9, 4.1)	< 0.001
Outcome 6: Recent Self-Harm Behaviours (SH) and Recent Marijuana Use (MU)			
No SH, No MU	1.0	-	-
SH Only	1.0	3.5 (2.4, 5.0)	< 0.001
MU Only	1.0	1.3 (1.0, 1.8)	0.08
SH and MU	1.0	6.0 (4.1, 8.8)	< 0.001
Outcome 7: Recent Suicidal Behaviour (SB^8^) and Recent Cigarette Use (CU)			
No SB, No CU	1.0	-	-
SB Only	1.0	3.3 (2.3, 4.9)	< 0.001
CU Only	1.0	1.2 (0.9, 1.6)	0.3
SB and CU	1.0	5.3 (3.7, 7.6)	< 0.001
Outcome 8: Recent Suicidal Behaviour (SB) and Recent Alcohol Use (AU)			
No SB, No AU	1.0	-	-
SB Only	1.0	1.8 (1.5, 2.2)	< 0.001
AU Only	1.0	0.7 (0.5, 0.9)	0.1
SB and AU	1.0	2.8 (1.9, 4.0)	< 0.001
Outcome 9: Recent Suicidal Behaviour (SB) and Recent Marijuana Use (MU)			
No SB, No MU	1.0	-	-
SB Only	1.0	3.7 (2.6, 5.3)	< 0.001
MU Only	1.0	1.5 (1.1, 2.0)	0.01
SB and MU	1.0	5.4 (3.7, 7.9)	< 0.001

Adjusted multinomial odds ratio

95% confidence interval

High or very high levels of psychological distress as self-reported on the Kessler Psychological Distress 10-item Scale

Past 12-month cigarette use

Past 12-month alcohol use

Past 12-month marijuana use

Past 12-month self-harm ideation or attempts

Past 12-month suicidal ideation, planning or attempts

Sensitivity analyses comprising unadjusted models testing crude associations differences in co-occurring mental ill-health and substance use outcomes by gender, sexuality, and sexuality diversity are presented in Supplementary Material B.

As shown in Table 4 below, trans people reported significantly increased odds of co-occurring mental ill-health and substance use in four out of 12 instances, specifically high or very high levels of psychological distress and recent cigarette use; self-harm thoughts/behaviours and cigarette use; self-harm behaviours and marijuana use; and suicidal thoughts/behaviour and cigarette use (all p-values < 0.001).

**Table 4:** Associations between young people’s gender identities and co-occurring mental ill-health and substance use outcomes

	Gender identity		
	Cisgender	Trans	
	*N* (%)	*N* (%)	*p*-value
Outcome 1: High or very high levels of psychological distress (K10) and Recent Cigarette Use (CU)	348 (14.7)	12 (32.6)	< 0.001
Outcome 2: High or very high levels of psychological distress (K10) and Recent Alcohol Use (AU)	701 (29.1)	20 (54.8)	< 0.001
Outcome 3: High or very high levels of psychological distress (K10) and Recent Marijuana Use (MU)	288 (12.0)	$*$	< 0.001
Outcome 4: Recent Self-harm Behaviours (SH) and Recent Cigarette Use (CU)	176 (7.4)	*	< 0.001
Outcome 5: Recent Self-harm Behaviours (SH) and Recent Alcohol Use (AU)	300 (12.5)	15 (40.2)	< 0.001
Outcome 6: Recent Self-Harm Behaviours (SH) and Recent Marijuana Use (MU)	154 (6.4)	*	< 0.001
Outcome 7: Recent Suicidal Behaviour (SB) and Recent Cigarette Use (CU)	180 (7.6)	*	0.001
Outcome 8: Recent Suicidal Behaviour (SB) and Recent Alcohol Use (AU)	304 (12.6)	14 (36.6)	< 0.001
Outcome 9: Recent Suicidal Behaviour (SB) and Recent Marijuana Use (MU)	151 (6.3)	*	< 0.001

*In accordance with Longitudinal Study of Australian Children dataset governance rules, the authors have censored cell counts *≤* 10

## Discussion

This study is the first to utilise population-level, nationally representative data to estimate and examine the prevalence, distribution and co-occurrence of mental ill-health and substance use among LGBTQ + young people in Australia. This study is also the first to include a comparison of cisgender and heterosexual/non-sexuality diverse participants allowing for robust estimation of the magnitude of disparities in mental ill-health, substance use and their co-occurrence among LGBTQ + young people in Australia.

We found that LGBTQ + youth experience significantly more significantly higher levels of psychological distress and higher rates of self-harm and suicidal thoughts/behaviours than their cisgender, heterosexual, and non-sexuality diverse peers, consistent with current literature [1, 2, 45]. A key finding of the present study was that self-harm behaviour and suicidal thoughts/behaviour disparities were most pronounced among trans young people. Trans young people were 4.9 times as likely than their cisgender peers to engage in self-harm behaviours, and 4.8 times as likely to engage in suicidal thoughts/behaviours. These estimates are lower than in previous research among trans young people in Australia, which may be accounted for by our use of population-representative sampling and inclusion of cisgender comparators [1, 2]. Moreover, our operationalisation of self-harm and suicidal behaviours rather than strictly ‘attempts’ is important because these other behaviours (e.g., ideation) are significant predictors of future self-harm and suicide attempts among young people and hence represent important targets for prevention and treatment [46]. Though the magnitudes of these odds ratios are particularly noteworthy, it is important to note the small sample size of trans people albeit representative [47] may bias these effect estimates [48]. These findings are a valuable advancement of the literature, given that research on the mental health of LGBTQ + young people often focuses exclusively on sexuality diverse young people [49]. Future policy and programs aimed at promoting the mental health of LGBTQ + young people should prioritise resource allocation toward trans young people and address their unmet, distinct needs given their disproportionate burden of mental ill-health.

This study found that sexuality diverse young people are significantly more likely than their non-sexuality diverse peers to report recent cigarette use and recent marijuana use, with these disparities higher among sexuality diverse females relative to non-sexuality diverse females. Though difficult to compare due to differing timepoints assessed, our findings that over two in five (43%) of sexuality diverse young people report using cigarettes in the preceding 12 months is more than double the one in five (20%) of LGBTQ + young people reporting ever having used a cigarette in the Writing Themselves in 4 study. Conversely, though discordant measures, our study found that nearly two in five (36%) sexuality diverse young people reported past 12-month marijuana use which is considerably higher than Writing Themselves In 4’s finding that roughly one in three (28%) LGBTQ + young people reported past six-month marijuana use [1]. It is important to note that recent substance use does not equate to substance use-related harms nor frequency and intensity of substance use. Hence, these findings of disparities in past 12-month cigarette use and past 12-month marijuana use warrant further attention into the embodied contexts of substance use among LGBTQ + young people. Such research should consider the positive, altruistic, and social aspects of substance use among LGBTQ + young people [18, 19] with the view to advancing LGBTQ + affirming models of substance use harm reduction. Future longitudinal research is also required, utilising additional population-level, nationally representative datasets to elucidate disparities in substance use among LGBTQ + young people specifically related to the age of initiation, frequency, and intensity of use through adolescence.

There is a dearth of epidemiological evidence regarding the burden and magnitude of co-occurring mental ill-health and substance use among LGBTQ + young people. Addressing this research gap, our study found that LGBTQ + youth in our sample experienced significantly higher rates of co-occurring mental ill-health and substance use than their cisgender, heterosexual, and non-sexuality diverse peers. Specifically, sexuality diverse young people were found to experience significantly higher rates of psychological distress, self-harm behaviours, and suicidal behaviours in co-occurrence with cigarette and marijuana use. While this result converges with and extends previous research finding that sexuality diverse young people are at greater odds of co-occurring depressive symptoms and substance use disorders [28], results from many previous studies are largely incomparable insofar as they have utilised samples spanning younger adolescents and adults, or examined mental ill-health and substance use separately [25, 28, 50]. Researchers and prevention practitioners alike would do well to consider harnessing ‘combined’ models of prevention for co-occurring mental ill-health and substance use [51] among LGBTQ + young people, an approach to prevention with demonstrated benefits for concurrently preventing mental ill-health and substance use and related harms during adolescence through early adulthood [52]. Adapting and evaluating prevention efforts to target co-occurring mental ill-health and substance use is critical for addressing the disproportionate burden, morbidity and mortality of these public health issues among LGBTQ + young people [53].

Conversely, our analyses indicated that trans young people were at increased odds of experiencing psychological distress, self-harm behaviours, and suicidal behaviours in co-occurrence with cigarette use and marijuana use. While there is a paucity of research investigating moderating factors between gender and substance use [54], research suggests trans young people often receive less family acceptance and social support compared with their sexuality diverse peers [55]. Hence our findings may be explained by previous research suggesting substance use among gender diverse young people may be a coping strategy in response to unique gender minority stressors [56]. These stressors, including heterosexist stigma and discrimination, are prevalent [5, 49] and bear deleterious mental ill-health effects for trans young people [8]. Future research should investigate the interconnected relationship between gender minority stressors and co-occurring mental ill-health and substance among trans young people and investigate modifiable risk and protective factors suitable for prevention and early intervention targeting.

Our observation of higher rates of mental ill-health outcomes among sexuality diverse young females relative to sexuality diverse young males aligns with current literature finding that young females experience higher rates of mental ill-health relative to young men [29]. Further community-engaged public health research and activities must investigate sexuality diverse young females and non-binary people’s experiences of mental ill-health and assess and address current unmet needs.

This study should be interpreted with consideration of strengths and limitations. Strengths included our use of a large, population-level, nationally representative cohort study; use of sample weighting and, where applicable, adjustment for geographical postcode-level clustering; separation of trans young people from sexuality diverse participants; inclusion of sexuality diverse people identified through sexual attraction and sexual behaviour items in addition to the sexual identity item; and systematic, rigorous approach to quantifying disparities in co-occurring mental ill-health and substance use among LGBTQ + young people. On the other hand, the small number of trans participants is a limitation of the present study, and results should be interpreted cautiously. The proportion of trans participants in our overall sample, however, is congruent with previous research estimating the number of trans people in Australia and worldwide (between 0.5 and 2.7%) [57, 58]. Furthermore, the demographic characteristics of trans and cisgender participants, as shown in Table 1, are relatively comparable. The cross-sectional nature of the present study is another limitation in that longitudinal trend analysis is required to identify the true burden of mental ill-health, substance use, and their co-occurrence among LGBTQ + young people. This paper represents the first step towards addressing this significant research gap; further research will analyse longitudinal trends and patterns of mental ill-health, substance use, and co-occurrence among LGBTQ + young people in Australia.

In summation, this study is the first to provide population-level, nationally representative epidemiological evidence highlighting the significant, deleterious burden and magnitude of mental ill-health and substance use disparities faced by LGBTQ + young people across Australia. Urgent public health attention is required to remediate those mental ill-health disparities, largest among trans young people, and those co-occurring mental ill-health and substance use inequities, largest among sexuality diverse young people. This paper also issues a call to action for public health researchers involved in designing, curating, analysing, and reporting large, population-based datasets to include adequate gender and sexuality indicators. Accurate, high-quality data is key for driving evidence-based, culturally safe [59] policy and practice to prevent and reduce mental ill-health and substance use among LGBTQ + young people in Australia.

## Electronic supplementary material

Below is the link to the electronic supplementary material.

Supplementary Material 1

Supplementary Material 2
