# Supplementary material for: Prevalence, distribution, and inequitable co-occurrence of mental ill-health and substance use among gender and sexuality diverse young people in Australia: epidemiological findings from a population-based cohort study
Source: Soc Psychiatry Psychiatr Epidemiol. 2024 Jul 23;59(12):2323–37. doi: 10.1007/s00127-024-02714-1 (PMC11522107; doi:10.1007/s00127-024-02714-1)
Supplement: Supplementary file 1 — Supplementary Material 1 [file 127_2024_2714_MOESM1_ESM.docx]

**Appendix A. STROBE Statement—checklist of items that should be included in reports of observational studies**

|  | Item No. | Recommendation | Page  No. | Relevant text from manuscript |
| --- | --- | --- | --- | --- |
| **Title and abstract** | 1 | (*a*) Indicate the study’s design with a commonly used term in the title or the abstract | 1 | ‘a population-based cohort study’ |
|  |  | (*b*) Provide in the abstract an informative and balanced summary of what was done and what was found | 3-4 | We utilised Wave 8 (2018) data from the Longitudinal Study of Australian Children (N=3037, M_age_ = 18.4) collected via an assessment protocol comprising interviews, direct observations, and assessments (on average 60 minutes per survey occasion). Weighted prevalence ratios and logistic regression models adjusted for demographic confounders were used to estimate the prevalence and distribution of mental ill-health (psychological distress, past 12-month self-harm thoughts and behaviours, past 12-month suicidal ideation, planning, attempt/s) and substance use outcomes (past 12-month cigarette, alcohol, and marijuana use) across gender identity (trans vs cisgender), sexuality (gay/lesbian, bisexual, queer [those identifying with an ‘other’ sexuality identity that is not ‘gay’, ‘lesbian’, ‘bisexual’, or ‘heterosexual’] vs heterosexual) and sexuality diversity status (sexuality diverse vs heterosexual) subgroups. Sex-stratified prevalence rates and accompanying adjusted logistic regression models were also used to assess mental ill-health and substance use disparities by sexuality diversity status. Adjusted multinominal logistic regression models were used to test disparities in co-occurring outcomes by sexuality identity) sexuality status sub-groups, and Fisher’s Exact Test of Independence for co-occurring disparities by gender identity (due to small sample size). All analyses used Wave 8 sample weights and adjusted for postcode-level clustering.  Among gender and sexuality diverse participants, 59% - 64% reported high or very high levels of psychological distress, 28% - 46% reported past 12-month self-harm ideation or attempts, and 26% - 46% reported past 12-month suicidal ideation, planning, or behaviour. We found significant disparities in high/very high levels of psychological distress, self-harm behaviours and suicidal behaviours among trans participants (adjusted odds ratios (aORs) ranged from 3.5-5.5) and sexuality diverse participants (aORs ranged from 3.5-3.9), compared with cisgender and heterosexual participants, respectively. Highest disparities in any past 12-month self-harm and suicidal behaviours appeared most pronounced among trans participants and queer participants compared with their cisgender, heterosexual counterparts. Minor differences by sex among sexuality diverse participants were observed for select mental ill-health outcomes. Sexuality diverse participants, and particularly sexuality diverse females, were significantly more likely to report past 12-month cigarette use and past 12-month marijuana use (adjusted odds ratio (aORs) ranging 1.4-1.6). Trans young people were at significantly elevated risk of mental ill-health in co-occurrence with cigarette and marijuana use compared with their cisgender peers (Fisher’s Exact Test of Independence p<0.05 for all), whereas sexuality diverse young people were at greater risk of co-occurring mental ill-health and cigarette co-use and marijuana co-use, compared with their non-sexuality diverse peers (adjusted multinomial odds ratios (aMORs) ranging 2.2-6.0). |
| Introduction | | | |  |
| Background/rationale | 2 | Explain the scientific background and rationale for the investigation being reported | 1-7 | While many gender and sexuality diverse (LGBTQ+) young people live healthy, fulfilled lives, higher rates of mental ill-health among LGBTQ+ young people relative to their cisgender and heterosexual peers have been documented in Australia and worldwide.^1-5^ For example, the Writing Themselves In 4 survey of 6,418 LGBTQ+ young people aged 14-21 in Australia found over 80% of participants reported high or very high levels of psychological distress, over 40% reported past 12-month self-harm, and up to 77% reported past 12-month suicidal ideation, thoughts or attempts.^1^ However, these estimates have significant heterogeneity across LGBTQ+ subgroups. The Trans Pathways survey of 859 trans, non-binary, and gender diverse (henceforth respectfully referred to with the umbrella term, ‘trans’) young people in Australia found approximately 75% of trans young people reported a depression diagnosis; 80% reported having ever self-harmed, and 48% reported attempting suicide at some stage in their lifetime.^2, 6^ Similar rates of mental ill-health among LGBTQ+ young people have been reported in other jurisdictions worldwide, including a disproportionately higher burden among trans young people.^5, 7^ A significant limitation of many of these prevalence studies, however, is the lack of population-level representativeness due to the use of convenience sampling methods, inadequate sample size/statistical power, the conflation of gender and sexuality, or lack of a cisgender and/or heterosexual comparison group. Accurate, population-based research is critical for understanding and preventing mental ill-health and related costs across the lifespan.  LGBTQ+ young people are also at increased risk of substance use relative to their cisgender and heterosexual peers.^8^ Research shows that substance use is more prevalent^9^ and initiated at earlier ages^10^ among LGBTQ+ young people relative to cisgender and heterosexual young people, particularly cigarette, alcohol and marijuana use.^11-14^ This warrants significant public health attention because mental ill-health and substance use typically emerge and co-occur during adolescence and share common, interacting and compounding risk factors.^15-17^ It is important to note that substance use in and of itself does not equate to substance use-related harms. Accordingly, past studies have found that many LGBTQ+ young people engage in substance use for altruistic, social, and community-seeking motivations.^18, 19^ Notwithstanding this, compared with individual occurrences, co-occurring mental ill-health and substance use bears significantly higher burden and morbidity and requires different strategies for prevention, early intervention and treatment.^20, 21^ Moreover, the risk factors which predispose co-occurrence may also create other vulnerabilities, including sexual risk-taking and adolescent behaviour problems.^22^ Despite the significant burden of mental ill-health and substance use among LGBTQ+ young people, current literature is siloed and does not consider the prevalence and distribution of co-occurring mental ill-health and substance use among LGBTQ+ young people.^23^ Scarce available literature regarding co-occurring mental ill-health and substance use among LGBTQ+ people focuses predominantly on sexuality diverse groups^24^ and LGBTQ+ adults.^25-27^ A recent study using national cohort data of LGBTQ+ young adults in the United States was conducted to estimate the prevalence of co-occurring depressive symptoms and probable substance use disorders; however, this study was limited to LGBTQ+ people aged 20-35 and did not stratify results by participants’ ages.^28^  Further research into co-occurring mental ill-health and substance use should estimate prevalence among LGBTQ+ young people of high school-leaving age (i.e., 18 years), typically associated with the onset and peak of substance use-related harms in mainstream populations.^29^ Whereas previous research has utilised disorder-level measures of co-occurring mental ill-health and substance use among LGBTQ+ people, there is scarce literature that utilises non-specific or transdiagnostic psychiatric screening tools and self-report measures of frequency of substance use.^30^ Screening and frequency-based tools more comprehensively capture substance use harms among this population because substance use during adolescence generally is associated with significant neurodevelopment harms and is a significant risk factor for developing and onset substance use disorders during adulthood.^21^ Lastly, current literature on co-occurring adolescent mental health and substance use disorders does not include self-harm and suicidal behaviours in the context of mental ill-health.^24, 28^ Self-harm and suicidal behaviours disproportionately affect LGBTQ+ young people^31, 32^ and are significantly associated with substance use.^33^ To our knowledge, little to no research has been conducted to investigate the potential co-occurrence of self-harm and suicidal behaviours with substance use among LGBTQ+ young people.  Over the past two decades, a significant body of high-quality, LGBTQ+ communities-engaged research has emerged, providing vital insights into the burden, magnitude and nature of mental ill-health and substance use among LGBTQ+ young people in Australia, forming a vital evidence basis for real-life public health policy and practice change for LGBTQ+ communities. These include the Writing Themselves In^1^, Trans Pathways^2^, Growing Up Queer^34^, The First Australian National Trans Mental Health^35^, and the From Blues to Rainbows^36^ studies. A significant limitation of many of these prevalence studies, however, is the lack of population-level representativeness due to the use of convenience sampling methods, inadequate sample size/statistical power, the conflation of gender and sexuality, or lack of a cisgender and/or heterosexual comparison group. Accurate, population-based research is critical for understanding and preventing mental ill-health and related costs across the lifespan. |
| Objectives | 3 | State specific objectives, including any prespecified hypotheses | 7-8 | The present study aimed to: (1) estimate the prevalence of mental ill-health and substance use among LGBTQ+ young people; and (2) compare differences in mental ill-health, substance use, and co-occurring mental ill-health and substance use outcomes between trans vs cisgender and sexuality diverse vs heterosexual participants in a population-level, nationally representative sample in Australia. |
| Methods | | | |  |
| Study design | 4 | Present key elements of study design early in the paper | 8 | The Longitudinal Study of Australian Children (LSAC) is a cross-sequential study comprising two 12-month age cohorts (B cohort, infants aged 0-1 years, and K cohort, children aged 4-5 years old when the study began). This study used data from Wave 8 of the K cohort, who were born between March 1999 and February 2000 and were followed up in 2018 when they were aged 17-19. The Wave 8 response rate was approximately 77.3%, and Wave 1 retention rate was approximately 61.1%.^37^ Full detail regarding using a non-probability-based selection of participations via geographically representative postcode sampling with a homogenous probability of selection is published elsewhere.^38^ LSAC methodology and survey content have received ethical review and approval by the Australian Institute of Family Studies Ethics Committee. |
| Setting | 5 | Describe the setting, locations, and relevant dates, including periods of recruitment, exposure, follow-up, and data collection | 3, 8 | We utilised Wave 8 (2018) data from the Longitudinal Study of Australian Children (N=3037, M_age_ = 18.4) collected via an assessment protocol comprising interviews, direct observations, and assessments (on average 60 minutes per survey occasion).  The Longitudinal Study of Australian Children (LSAC) is a cross-sequential study comprising two 12-month age cohorts (B cohort, infants aged 0-1 years, and K cohort, children aged 4-5 years old when the study began). This study used data from Wave 8 of the K cohort, who were born between March 1999 and February 2000 and were followed up in 2018 when they were aged 17-19. The Wave 8 response rate was approximately 77.3%, and Wave 1 retention rate was approximately 61.1%.^37^ Full detail regarding using a non-probability-based selection of participations via geographically representative postcode sampling with a homogenous probability of selection is published elsewhere.^38^ LSAC methodology and survey content have received ethical review and approval by the Australian Institute of Family Studies Ethics Committee. |
| Participants | 6 | *Cross-sectional study*—Give the eligibility criteria, and the sources and methods of selection of participants |  | The LSAC sample was selected from the Medicare Australia enrolments database, Australia’s most comprehensive population database, particularly of young children.^38^ A two-stage clustered design was employed wherein 311 geographic postcodes were randomly selected, following which, children were subsequently randomly selected within each postcode.^38^ Stratification was utilised to ensure that numbers of children selected were roughly proportionate to the total number of children within each Australian state/territory, capital city districts and broader regional surrounding areas.^38^ This method of accounting for the number of children in each postcode meant that potentially participants across Australia had an approximately equal chance of selection (approximately one in 25).^38^ Full detail regarding using a non-probability-based selection of participations via geographically representative postcode sampling with a homogenous probability of selection is published elsewhere.^38^ |
|  |  |  |  |  |
| Variables | 7 | Clearly define all outcomes, exposures, predictors, potential confounders, and effect modifiers. Give diagnostic criteria, if applicable | 13, 14 | **Sexuality identity (individual-level sexuality diversity).** Participants were asked, “Which of the following categories best describes how you think of yourself” with options including ‘Heterosexual or straight’, ‘Gay or lesbian’, ‘Bisexual’, ‘Other’, and ‘Don’t know’.  **Sexuality diversity (group-level sexuality diversity).** A ‘Sexuality Diversity’ variable was computed where Sexuality Diversity included those who indicated that their sexuality was ‘Gay or Lesbian’, ‘Bisexual’, ‘Other’, or ‘Don’t know’. Additionally, a sexual attraction item was asked of participants: ‘Which of these statements best describes your sexual feelings at this time in your life?’ with possible responses including ‘Only attracted to females’, ‘Mostly attracted to females’, ‘I’m equally attracted to females and males’, ‘Mostly attracted to males’, ‘Only attracted to males’, ‘Never felt attracted to anybody at all’, and ‘Unsure’. Male participants who responded that they were only, mostly, or equally attracted to males, female participants who responded that they were only, mostly, or equally attracted to females, and participants who responded that they had never felt attracted to anybody or were unsure about their sexual feelings, were coded as ‘Sexuality Diverse’. A third item was used for this Sexuality Diversity variable assessing the sex of participants’ past sexual partners: ‘Which of the following options best describes who you have had sex with during the last 12 months?’ Responses were ‘Only with males’, ‘Only with females’, and ‘With both males and females’. Male participants who reported that they had had sex only with males, female participants who reported that they had had sex only with females, and participants who responded that they had had sex with both males and females, were also coded as ‘Sexuality Diverse’. Those who did not meet these criteria were coded as ‘Non-Sexuality Diverse’.  **Gender identity**. Participants were asked about their Sex (Male or Female) and Gender Identity (Male; Female; Transgender, male to female; Transgender, female to male; Genderqueer; and Other). Trans people were participants who explicitly identified as transgender, and those who indicated Gender Identity different from Sex. Participants with concordant Gender Identity and Sex responses were classified as Cisgender.  **Mental ill-health.** Past Australia-specific research examining distributions of mental ill-health among LGBTQA+ young people has concentrated on psychological distress^1^ and experiences of self-harm^1, 6^ and suicidality^1, 6^ thus the researchers sought to analyse those identical or similar concordant measures of psychological distress, self-harm, and suicidality within LSAC to enable cross-cohort comparisons.  Psychological distress. The 10-item Kessler Psychological Distress Scale (K10) was administered to participants to capture past four-week non-specific psychological distress.^39^ A scale score is calculated by summing scores for each item with higher scores indicating greater psychological distress. Previous research has shown that the K10 measure possesses sound factor structure and predictive validity in Australian child and adolescent populations.^40^ As defined by the Australian Bureau of Statistics, scores >22 indicate high or very high levels of psychological distress.^39, 41^  Self-harm thoughts and behaviour. Participants indicated ‘yes’ or ‘no’ to whether, in the past 12 months they had ‘thought about hurting (themselves) on purpose in any way?’ or ‘hurt (themselves) on purpose in any way?’.  Suicidal thoughts and behaviours. Participants were asked (yes/no) whether, during the past 12 months, they had ‘ever seriously consider(ed) attempting suicide?’ or ‘made a plan about how (they) would attempt suicide?’ Participants were also asked, ‘During the past 12 months, how many times did you actually attempt suicide? (0 times, 1 time, 2 or 3 times, 4 or 5 times, 6 or more times). Suicide attempts were categorised and dichotomised where >1 indicated a past 12-month suicide attempt, and 0 indicated no past 12-month suicide attempt.  **Substance use.**  Past 12-month cigarette, alcohol, and marijuana use. Six separate items assessed participants' ever use and past 12-month use of cigarettes, alcohol, and marijuana (yes/no items). For each substance use outcome, participants were coded as indicating past 12-month use if they reported ever use *and* past 12-month use for a given substance. Participants were coded as not reporting past 12-month use if they indicated they had never used a given substance *or* had not used that substance use in the past 12-months. Due to low sample sizes, the researchers were unable to analyse responses to other drugs, such as hallucinogens, methamphetamine, and opioids. Similarly, low cell counts prevented the researchers from conducting gender and sexuality-specific sub-group analyses using past 4-week substance use, past 7-day substance, recent participation in hazardous drinking, and current smoking status. Notwithstanding this, much of the existing literature related to substance use among LGBTQ+ young people specifically (and not adults) in Australia focuses on the burden of cigarette use and alcohol consumption^1, 6^ and less about use of other ‘illicit drugs’^42^ hence the researchers were still able to make meaningful cross-cohort comparisons.  **Co-occurring mental ill-health and substance use.**  Utilising the same approach implemented in a previous study of co-occurring depressive symptoms and probable substance use disorders among LGBTQ+ people^28^, we created nine new variables for co-occurring mental ill-health and substance use:   1. *High/very high levels of psychological distress and past 12-month cigarette use*: (a) no past 4-week high or very high levels of psychological distress, no past 12-month cigarette use, (b) past 4-week high or very high levels of psychological distress only (c) past 12-month cigarette use only, (d) both past 4-week high or very high levels of psychological distress and past 12-month cigarette use. 2. *High/very high levels of psychological distress and past 12-month alcohol use*: (a) no past 4-week high or very high levels of psychological distress, no past 12-month alcohol use, (b) past 4-week high or very high levels of psychological distress only, (c) past 12-month alcohol use only, (d) both past 4-week high or very high levels of psychological distress and past 12-month alcohol use. 3. *High/very high levels of psychological distress and past 12-month marijuana use:* (a) no past 4-week high or very high levels of psychological distress, no past 12-month marijuana use, (b) past 4-week high or very high levels of psychological distress only, (c) past 12-month marijuana use only, (d) both past 4-week high or very high levels of psychological distress and past 12-month marijuana use. 4. *Past 12-month self-harm thoughts/behaviours and past 12-month cigarette use*: (a) no past 12-month self-harm ideation or attempts, no past 12-month cigarette use, (b) past 12-month self-harm ideation or attempts only, (c) past 12-month cigarette use only, (d) both past 12-month self-harm ideation or attempts and past 12-month cigarette use. 5. *Past 12-month self-harm thoughts/behaviours and past 12-month alcohol use*: (a) no past 12-month self-harm ideation or attempts, no past 12-month alcohol use, (b) past 12-month self-harm ideation or attempts only, (c) past 12-month alcohol use only, (d) both past 12-month self-harm ideation or attempts and past 12-month alcohol use. 6. *Past 12-month self-harm thoughts/behaviours and past 12-month marijuana use:* (a) no past 12-month self-harm ideation or attempts, no past 12-month marijuana use, (b) past 12-month self-harm ideation or attempts only, (c) past 12-month marijuana use only, (d) both past 12-month self-harm ideation or attempts and past 12-month marijuana use. 7. *Past 12-month suicidal thoughts/behaviours and past 12-month cigarette use:* (a) no past 12-month suicidal ideation, planning or attempts, no past 12-month cigarette use, (b) past 12-month suicidal ideation, planning or attempts only, (c) past 12-month cigarette use only, (d) both past 12-month suicidal ideation, planning or attempts and past 12-month cigarette use. 8. *Past 12-month suicidal thoughts/behaviours and past 12-month alcohol use:* (a) no past 12-month suicidal ideation, planning or attempts, no past 12-month alcohol use, (b) past 12-month suicidal ideation, planning or attempts only, (c) past 12-month alcohol use only, (d) both past 12-month suicidal ideation, planning or attempts and past 12-month alcohol use. 9. *Past 12-month suicidal thoughts/behaviours and past 12-month marijuana use:* (a) no past 12-month suicidal ideation, planning or attempts, no past 12-month marijuana use, (b) past 12-month suicidal ideation, planning or attempts only, (c) past 12-month marijuana use only, (d) both past 12-month suicidal ideation, planning or attempts and past 12-month marijuana use.   Multivariate logistic regression models controlling for age, sex, socio-economic status, and region of residence were used to calculate adjusted odds ratios to compare differences in prevalence of mental ill-health (high/very high level of psychological distress, past 12-month self-harm ideation and attempts, and past 12-month suicidal ideation, planning and attempts) and substance use (past 12-month cigarette use, alcohol use, and marijuana use) between: heterosexual participants and gay/lesbian, bisexual, and other-sexuality participants (categorised separately); non-sexuality-diverse and sexuality diverse participants; and trans and cisgender participants. For these logistic regression models, referent groups comprised heterosexual, non-sexuality-diverse, and cisgender participants, respectively. Sex-stratified weighted prevalence statistics and multivariate logistic regression models controlling for age, sex, socio-economic status, and region of residence were also computed to describe and test sex-varying differences in mental ill-health and substance use between sexuality diverse vs non-sexuality diverse participants. Multinomial logistic regression estimates controlling for age, sex, socio-economic status, and region of residence, were conducted to test for associations between sexuality diversity and co-occurring mental ill-health and substance use outcomes. |
| Data sources/ measurement | 8* | For each variable of interest, give sources of data and details of methods of assessment (measurement). Describe comparability of assessment methods if there is more than one group | 13 | **Sexuality identity (individual-level sexuality diversity).** Participants were asked, “Which of the following categories best describes how you think of yourself” with options including ‘Heterosexual or straight’, ‘Gay or lesbian’, ‘Bisexual’, ‘Other’, and ‘Don’t know’.  **Sexuality diversity (group-level sexuality diversity).** A ‘Sexuality Diversity’ variable was computed where Sexuality Diversity included those who indicated that their sexuality was ‘Gay or Lesbian’, ‘Bisexual’, ‘Other’, or ‘Don’t know’. Additionally, a sexual attraction item was asked of participants: ‘Which of these statements best describes your sexual feelings at this time in your life?’ with possible responses including ‘Only attracted to females’, ‘Mostly attracted to females’, ‘I’m equally attracted to females and males’, ‘Mostly attracted to males’, ‘Only attracted to males’, ‘Never felt attracted to anybody at all’, and ‘Unsure’. Male participants who responded that they were only, mostly, or equally attracted to males, female participants who responded that they were only, mostly, or equally attracted to females, and participants who responded that they had never felt attracted to anybody or were unsure about their sexual feelings, were coded as ‘Sexuality Diverse’. A third item was used for this Sexuality Diversity variable assessing the sex of participants’ past sexual partners: ‘Which of the following options best describes who you have had sex with during the last 12 months?’ Responses were ‘Only with males’, ‘Only with females’, and ‘With both males and females’. Male participants who reported that they had had sex only with males, female participants who reported that they had had sex only with females, and participants who responded that they had had sex with both males and females, were also coded as ‘Sexuality Diverse’. Those who did not meet these criteria were coded as ‘Non-Sexuality Diverse’.  **Gender identity**. Participants were asked about their Sex (Male or Female) and Gender Identity (Male; Female; Transgender, male to female; Transgender, female to male; Genderqueer; and Other). Trans people were participants who explicitly identified as transgender, and those who indicated Gender Identity different from Sex. Participants with concordant Gender Identity and Sex responses were classified as Cisgender.  **Mental ill-health.** Past Australia-specific research examining distributions of mental ill-health among LGBTQA+ young people has concentrated on psychological distress^1^ and experiences of self-harm^1, 6^ and suicidality^1, 6^ thus the researchers sought to analyse those identical or similar concordant measures of psychological distress, self-harm, and suicidality within LSAC to enable cross-cohort comparisons.  Psychological distress. The 10-item Kessler Psychological Distress Scale (K10) was administered to participants to capture past four-week non-specific psychological distress.^39^ A scale score is calculated by summing scores for each item with higher scores indicating greater psychological distress. Previous research has shown that the K10 measure possesses sound factor structure and predictive validity in Australian child and adolescent populations.^40^ As defined by the Australian Bureau of Statistics, scores >22 indicate high or very high levels of psychological distress.^39, 41^  Self-harm thoughts and behaviour. Participants indicated ‘yes’ or ‘no’ to whether, in the past 12 months they had ‘thought about hurting (themselves) on purpose in any way?’ or ‘hurt (themselves) on purpose in any way?’.  Suicidal thoughts and behaviours. Participants were asked (yes/no) whether, during the past 12 months, they had ‘ever seriously consider(ed) attempting suicide?’ or ‘made a plan about how (they) would attempt suicide?’ Participants were also asked, ‘During the past 12 months, how many times did you actually attempt suicide? (0 times, 1 time, 2 or 3 times, 4 or 5 times, 6 or more times). Suicide attempts were categorised and dichotomised where >1 indicated a past 12-month suicide attempt, and 0 indicated no past 12-month suicide attempt.  **Substance use.**  Past 12-month cigarette, alcohol, and marijuana use. Six separate items assessed participants' ever use and past 12-month use of cigarettes, alcohol, and marijuana (yes/no items). For each substance use outcome, participants were coded as indicating past 12-month use if they reported ever use *and* past 12-month use for a given substance. Participants were coded as not reporting past 12-month use if they indicated they had never used a given substance *or* had not used that substance use in the past 12-months. Due to low sample sizes, the researchers were unable to analyse responses to other drugs, such as hallucinogens, methamphetamine, and opioids. Similarly, low cell counts prevented the researchers from conducting gender and sexuality-specific sub-group analyses using past 4-week substance use, past 7-day substance, recent participation in hazardous drinking, and current smoking status. Notwithstanding this, much of the existing literature related to substance use among LGBTQ+ young people specifically (and not adults) in Australia focuses on the burden of cigarette use and alcohol consumption^1, 6^ and less about use of other ‘illicit drugs’^42^ hence the researchers were still able to make meaningful cross-cohort comparisons.  **Co-occurring mental ill-health and substance use.**  Utilising the same approach implemented in a previous study of co-occurring depressive symptoms and probable substance use disorders among LGBTQ+ people^28^, we created nine new variables for co-occurring mental ill-health and substance use:   1. *High/very high levels of psychological distress and past 12-month cigarette use*: (a) no past 4-week high or very high levels of psychological distress, no past 12-month cigarette use, (b) past 4-week high or very high levels of psychological distress only (c) past 12-month cigarette use only, (d) both past 4-week high or very high levels of psychological distress and past 12-month cigarette use. 2. *High/very high levels of psychological distress and past 12-month alcohol use*: (a) no past 4-week high or very high levels of psychological distress, no past 12-month alcohol use, (b) past 4-week high or very high levels of psychological distress only, (c) past 12-month alcohol use only, (d) both past 4-week high or very high levels of psychological distress and past 12-month alcohol use. 3. *High/very high levels of psychological distress and past 12-month marijuana use:* (a) no past 4-week high or very high levels of psychological distress, no past 12-month marijuana use, (b) past 4-week high or very high levels of psychological distress only, (c) past 12-month marijuana use only, (d) both past 4-week high or very high levels of psychological distress and past 12-month marijuana use. 4. *Past 12-month self-harm thoughts/behaviours and past 12-month cigarette use*: (a) no past 12-month self-harm ideation or attempts, no past 12-month cigarette use, (b) past 12-month self-harm ideation or attempts only, (c) past 12-month cigarette use only, (d) both past 12-month self-harm ideation or attempts and past 12-month cigarette use. 5. *Past 12-month self-harm thoughts/behaviours and past 12-month alcohol use*: (a) no past 12-month self-harm ideation or attempts, no past 12-month alcohol use, (b) past 12-month self-harm ideation or attempts only, (c) past 12-month alcohol use only, (d) both past 12-month self-harm ideation or attempts and past 12-month alcohol use. 6. *Past 12-month self-harm thoughts/behaviours and past 12-month marijuana use:* (a) no past 12-month self-harm ideation or attempts, no past 12-month marijuana use, (b) past 12-month self-harm ideation or attempts only, (c) past 12-month marijuana use only, (d) both past 12-month self-harm ideation or attempts and past 12-month marijuana use. 7. *Past 12-month suicidal thoughts/behaviours and past 12-month cigarette use:* (a) no past 12-month suicidal ideation, planning or attempts, no past 12-month cigarette use, (b) past 12-month suicidal ideation, planning or attempts only, (c) past 12-month cigarette use only, (d) both past 12-month suicidal ideation, planning or attempts and past 12-month cigarette use. 8. *Past 12-month suicidal thoughts/behaviours and past 12-month alcohol use:* (a) no past 12-month suicidal ideation, planning or attempts, no past 12-month alcohol use, (b) past 12-month suicidal ideation, planning or attempts only, (c) past 12-month alcohol use only, (d) both past 12-month suicidal ideation, planning or attempts and past 12-month alcohol use. 9. *Past 12-month suicidal thoughts/behaviours and past 12-month marijuana use:* (a) no past 12-month suicidal ideation, planning or attempts, no past 12-month marijuana use, (b) past 12-month suicidal ideation, planning or attempts only, (c) past 12-month marijuana use only, (d) both past 12-month suicidal ideation, planning or attempts and past 12-month marijuana use.   Multivariate logistic regression models controlling for age, sex, socio-economic status, and region of residence were used to calculate adjusted odds ratios to compare differences in prevalence of mental ill-health (high/very high level of psychological distress, past 12-month self-harm ideation and attempts, and past 12-month suicidal ideation, planning and attempts) and substance use (past 12-month cigarette use, alcohol use, and marijuana use) between: heterosexual participants and gay/lesbian, bisexual, and other-sexuality participants (categorised separately); non-sexuality-diverse and sexuality diverse participants; and trans and cisgender participants. For these logistic regression models, referent groups comprised heterosexual, non-sexuality-diverse, and cisgender participants, respectively. Sex-stratified weighted prevalence statistics and multivariate logistic regression models controlling for age, sex, socio-economic status, and region of residence were also computed to describe and test sex-varying differences in mental ill-health and substance use between sexuality diverse vs non-sexuality diverse participants. Multinomial logistic regression estimates controlling for age, sex, socio-economic status, and region of residence, were conducted to test for associations between sexuality diversity and co-occurring mental ill-health and substance use outcomes. |
| Bias | 9 | Describe any efforts to address potential sources of bias | 15 | Utilising multinomial logistic regression to analyse dependent variables with several, unordered categories instead of collapsing dependent variables into two mutually exclusive groups to utilise binary logistic regression, maintains higher statistical power, does not require the satisfaction of assumptions regarding normality, linearity, or homoscedasticity, and provides easy interpretability. Furthermore, multinominal logistic regression analysis assumes non-perfect separation, which is particularly salient to the present study given that many young people who do not have co-occurring mental ill-health and substance use may still have mental ill-health or substance use, rather than neither.  Due to the small sample size of trans participants, Fisher’s Exact Tests of Independence were performed to detect associations between participants’ gender identity and co-occurring mental ill-health and substance use outcomes.  Given the exploratory nature of this epidemiological work, a statistically significant threshold of p <0.05 was used for all models.  All generalised linear models, including logistic regression models, adjusted for postcode-level clustering.  Complete case analysis was also used for multinomial models testing co-occurring mental ill-health and substance use disparities by sexuality diversity, and Fisher’s Exact Tests of Independence for disparities by gender. |
| Study size | 10 | Explain how the study size was arrived at | 8 | The Longitudinal Study of Australian Children (LSAC) is a cross-sequential study comprising two 12-month age cohorts (B cohort, infants aged 0-1 years, and K cohort, children aged 4-5 years old when the study began). This study used data from Wave 8 of the K cohort, who were born between March 1999 and February 2000 and were followed up in 2018 when they were aged 17-19. The Wave 8 response rate was approximately 77.3%, and Wave 1 retention rate was approximately 61.1%.^37^ |

Continued on next page

| Quantitative variables | 11 | Explain how quantitative variables were handled in the analyses. If applicable, describe which groupings were chosen and why | 14-15 | For descriptive statistics, categorical variables were summarised with counts and proportions and continuous variables, with means and standard deviations. Generalised linear models were used to adjust for postcode-level clustering. Multivariate logistic regression models controlling for age, sex, socio-economic status, and region of residence were used to calculate adjusted odds ratios to compare differences in prevalence of mental ill-health (high/very high level of psychological distress, past 12-month self-harm ideation and attempts, and past 12-month suicidal ideation, planning and attempts) and substance use (past 12-month cigarette use, alcohol use, and marijuana use) between: heterosexual participants and gay/lesbian, bisexual, and other-sexuality participants (categorised separately); non-sexuality-diverse and sexuality diverse participants; and trans and cisgender participants. For these logistic regression models, referent groups comprised heterosexual, non-sexuality-diverse, and cisgender participants, respectively. Sex-stratified weighted prevalence statistics and multivariate logistic regression models controlling for age, sex, socio-economic status, and region of residence were also computed to describe and test sex-varying differences in mental ill-health and substance use between sexuality diverse vs non-sexuality diverse participants. Multinomial logistic regression estimates controlling for age, sex, socio-economic status, and region of residence, were conducted to test for associations between sexuality diversity and co-occurring mental ill-health and substance use outcomes. Utilising multinomial logistic regression to analyse dependent variables with several, unordered categories instead of collapsing dependent variables into two mutually exclusive groups to utilise binary logistic regression, maintains higher statistical power, does not require the satisfaction of assumptions regarding normality, linearity, or homoscedasticity, and provides easy interpretability. Furthermore, multinominal logistic regression analysis assumes non-perfect separation, which is particularly salient to the present study given that many young people who do not have co-occurring mental ill-health and substance use may still have mental ill-health or substance use, rather than neither. Due to the small sample size of trans participants, Fisher’s Exact Tests of Independence were performed to detect associations between participants’ gender identity and co-occurring mental ill-health and substance use outcomes. |
| --- | --- | --- | --- | --- |
| Statistical methods | 12 | (*a*) Describe all statistical methods, including those used to control for confounding | 14-15 | For descriptive statistics, categorical variables were summarised with counts and proportions and continuous variables, with means and standard deviations. Generalised linear models were used to adjust for postcode-level clustering. Multivariate logistic regression models controlling for age, sex, socio-economic status, and region of residence were used to calculate adjusted odds ratios to compare differences in prevalence of mental ill-health (high/very high level of psychological distress, past 12-month self-harm ideation and attempts, and past 12-month suicidal ideation, planning and attempts) and substance use (past 12-month cigarette use, alcohol use, and marijuana use) between: heterosexual participants and gay/lesbian, bisexual, and other-sexuality participants (categorised separately); non-sexuality-diverse and sexuality diverse participants; and trans and cisgender participants. For these logistic regression models, referent groups comprised heterosexual, non-sexuality-diverse, and cisgender participants, respectively. Sex-stratified weighted prevalence statistics and multivariate logistic regression models controlling for age, sex, socio-economic status, and region of residence were also computed to describe and test sex-varying differences in mental ill-health and substance use between sexuality diverse vs non-sexuality diverse participants. Multinomial logistic regression estimates controlling for age, sex, socio-economic status, and region of residence, were conducted to test for associations between sexuality diversity and co-occurring mental ill-health and substance use outcomes. Utilising multinomial logistic regression to analyse dependent variables with several, unordered categories instead of collapsing dependent variables into two mutually exclusive groups to utilise binary logistic regression, maintains higher statistical power, does not require the satisfaction of assumptions regarding normality, linearity, or homoscedasticity, and provides easy interpretability. Furthermore, multinominal logistic regression analysis assumes non-perfect separation, which is particularly salient to the present study given that many young people who do not have co-occurring mental ill-health and substance use may still have mental ill-health or substance use, rather than neither. Due to the small sample size of trans participants, Fisher’s Exact Tests of Independence were performed to detect associations between participants’ gender identity and co-occurring mental ill-health and substance use outcomes.  Given the exploratory nature of this epidemiological work, a statistically significant threshold of p <0.05 was used for all models. All analyses utilised Wave 8 composite sample weights to correct for participant attrition over waves and to realign samples with the original probability sample design of the study when K-cohort participants were 4-5 years old. All generalised linear models, including logistic regression models, adjusted for postcode-level clustering. All statistical analyses were conducted using statistical software, RStudio (Version 4.2.2). |
|  |  | (*b*) Describe any methods used to examine subgroups and interactions | 14 | Multivariate logistic regression models controlling for age, sex, socio-economic status, and region of residence were used to calculate adjusted odds ratios to compare differences in prevalence of mental ill-health (high/very high level of psychological distress, past 12-month self-harm ideation and attempts, and past 12-month suicidal ideation, planning and attempts) and substance use (past 12-month cigarette use, alcohol use, and marijuana use) between: heterosexual participants and gay/lesbian, bisexual, and other-sexuality participants (categorised separately); non-sexuality-diverse and sexuality diverse participants; and trans and cisgender participants. For these logistic regression models, referent groups comprised heterosexual, non-sexuality-diverse, and cisgender participants, respectively. Sex-stratified weighted prevalence statistics and multivariate logistic regression models controlling for age, sex, socio-economic status, and region of residence were also computed to describe and test sex-varying differences in mental ill-health and substance use between sexuality diverse vs non-sexuality diverse participants. Multinomial logistic regression estimates controlling for age, sex, socio-economic status, and region of residence, were conducted to test for associations between sexuality diversity and co-occurring mental ill-health and substance use outcomes. |
|  |  | (*c*) Explain how missing data were addressed | 15 | There was <13% missing data affecting all mental ill-health outcomes assessed in this study (psychological distress, self-harm thoughts and behaviours, suicide thoughts and behaviours). However further inspection stratifying these measures by gender and sexuality found that <1% of participants reporting missing data for any of these mental ill-health outcomes also reported gender and sexuality data. Hence, to handle this missing data, complete case analysis was used excluding participants who did not record complete data on gender and sexuality as well as mental ill-health outcomes. To further mitigate impacts of missing data, complete case analysis was also used for past 12-month substance use outcomes wherein eligible participants included in the substance use analyses were required to record valid responses (yes or no) to both ‘ever use’ *and* ‘past 12-month use’ substance use items. Complete case analysis was also used for multinomial models testing co-occurring mental ill-health and substance use disparities by sexuality diversity, and Fisher’s Exact Tests of Independence for disparities by gender. |
|  |  | *Cross-sectional study*—If applicable, describe analytical methods taking account of sampling strategy | 15 | . All generalised linear models, including logistic regression models, adjusted for postcode-level clustering. |
|  |  | (*e*) Describe any sensitivity analyses | 14 | Sex-stratified weighted prevalence statistics and multivariate logistic regression models controlling for age, sex, socio-economic status, and region of residence were also computed to describe and test sex-varying differences in mental ill-health and substance use between sexuality diverse vs non-sexuality diverse participants. |
| Results | | | | |
| Participants | 13* | (a) Report numbers of individuals at each stage of study—eg numbers potentially eligible, examined for eligibility, confirmed eligible, included in the study, completing follow-up, and analysed | 8 | The Wave 8 response rate was approximately 77.3%, and Wave 1 retention rate was approximately 61.1%.^37^ |
|  |  | (b) Give reasons for non-participation at each stage |  | As the LSAC dataset is administered by the Australian Institute of Family Studies, detailed and public information regarding participants’ non-participation at each stage is unavailable. |
|  |  | (c) Consider use of a flow diagram |  | Ibid |
| Descriptive data | 14* | (a) Give characteristics of study participants (eg demographic, clinical, social) and information on exposures and potential confounders | 16 | Among the 3,037 K-cohort young people participating in Wave 8 of LSAC data collection (M_age_ = 18.4, SD_age_ = 0.5), 2,261 (87.6%) identified as heterosexual/straight, 56 (2.2%) gay/lesbian, 225 (8.7%) bisexual, and 39 (1.5%) ‘other’ sexuality (henceforth referred to respectfully as “those with other sexualities”). At an overall group-level categorisation of sexuality diversity, 402 participants (14.3%) were sexuality diverse and 2,211 participants (85.6%) were non-sexuality diverse. Of the total sample, there were 36 (1.4%) trans participants and 2,619 (98.6%) cisgender participants. |
|  |  | (b) Indicate number of participants with missing data for each variable of interest | 15 | There was <13% missing data affecting all mental ill-health outcomes assessed in this study (psychological distress, self-harm thoughts and behaviours, suicide thoughts and behaviours). However further inspection stratifying these measures by gender and sexuality found that <1% of participants reporting missing data for any of these mental ill-health outcomes also reported gender and sexuality data. Hence, to handle this missing data, complete case analysis was used excluding participants who did not record complete data on gender and sexuality as well as mental ill-health outcomes. To further mitigate impacts of missing data, complete case analysis was also used for past 12-month substance use outcomes wherein eligible participants included in the substance use analyses were required to record valid responses (yes or no) to both ‘ever use’ *and* ‘past 12-month use’ substance use items. Complete case analysis was also used for multinomial models testing co-occurring mental ill-health and substance use disparities by sexuality diversity, and Fisher’s Exact Tests of Independence for disparities by gender. |
|  |  |  |  |  |
| Outcome data | 15* |  |  |  |
|  |  |  |  |  |
|  |  | *Cross-sectional study—*Report numbers of outcome events or summary measures | 16 | Among the 3,037 K-cohort young people participating in Wave 8 of LSAC data collection (M_age_ = 18.4, SD_age_ = 0.5), 2,261 (87.6%) identified as heterosexual/straight, 56 (2.2%) gay/lesbian, 225 (8.7%) bisexual, and 39 (1.5%) ‘other’ sexuality (henceforth referred to respectfully as “those with other sexualities”). At an overall group-level categorisation of sexuality diversity, 402 participants (14.3%) were sexuality diverse and 2,211 participants (85.6%) were non-sexuality diverse. Of the total sample, there were 36 (1.4%) trans participants and 2,619 (98.6%) cisgender participants. More detail regarding the demographic characteristics of these LGBTQ+ groups and their heterosexual (identity-level), non-sexuality diverse (group-level), and cisgender comparators is available in Table 1. |
| Main results | 16 | (*a*) Give unadjusted estimates and, if applicable, confounder-adjusted estimates and their precision (eg, 95% confidence interval). Make clear which confounders were adjusted for and why they were included |  | Unadjusted estimates for models detailed above are presented in **Supplementary Material B**. Age was selected as covariates given the exponential rise of mental ill-health and substance use during adolescence; just under half (48.4%) of all mental disorders onset before age 18.^43^ Sex was also identified as a covariate given past research has shown that the co-occurrence of mental ill-health and substance use among LGBTQ+ young people differs by sex presumed at birth.^24^ Lastly, socio-economic status and region of residence were included as model covariates given their interaction and association with mental ill-health and substance use among young people.^44^ |
|  |  | (*b*) Report category boundaries when continuous variables were categorized | 19 | Namely, sexuality diverse young people were at increased odds of co-occurring high or very high levels of psychological distress and substance use, particularly cigarette use (adjusted multinomial odds ratio (aMOR) = 4.7, 95% CI: 3.4-6.5, p <0.001); alcohol use (aMOR = 2.2, 95% CI: 1.7-2.8, p <0.001); and marijuana use (aMOR = 5.3, 95% CI: 3.8-7.4, p <0.001), after controlling for age, sex, socio-economic status, and region of residence.  As shown in Table 4 below, trans people reported significantly increased odds of co-occurring mental ill-health and substance use in four out of 12 instances, specifically high or very high levels of psychological distress and recent cigarette use; self-harm thoughts/behaviours and cigarette use; self-harm behaviours and marijuana use; and suicidal thoughts/behaviour and cigarette use (all p-values <0.001). |
|  |  |  |  |  |

Continued on next page

| Other analyses | 17 | Report other analyses done—eg analyses of subgroups and interactions, and sensitivity analyses | 18 | Sensitivity analyses comprising unadjusted models testing crude associations differences in mental ill-health by gender, sexuality, and sexuality diversity are presented in Appendix B.  Sensitivity analyses comprising unadjusted models testing crude associations differences in substance use by gender, sexuality, and sexuality diversity are presented in Appendix B.  Sensitivity analyses comprising unadjusted models testing crude associations differences in co-occurring mental ill-health and substance use outcomes by gender, sexuality, and sexuality diversity are presented in Appendix B. |
| --- | --- | --- | --- | --- |
| Discussion | | | | |
| Key results | 18 | Summarise key results with reference to study objectives | 21-23 | We found that LGBTQ+ youth experience significantly more significantly higher levels of psychological distress and higher rates of self-harm and suicidal thoughts/behaviours than their cisgender, heterosexual, and non-sexuality diverse peers, consistent with current literature.^1, 2, 43^  This study found that sexuality diverse young people are significantly more likely than their non-sexuality diverse peers to report recent cigarette use and recent marijuana use, with these disparities higher among sexuality diverse females relative to non-sexuality diverse females.  Addressing this research gap, our study found that LGBTQ+ youth in our sample experienced significantly higher rates of co-occurring mental ill-health and substance use than their cisgender, heterosexual, and non-sexuality diverse peers. Specifically, sexuality diverse young people were found to experience significantly higher rates of psychological distress, self-harm behaviours, and suicidal behaviours in co-occurrence with cigarette and marijuana use.  Conversely, our analyses indicated that trans young people were at increased odds of experiencing psychological distress, self-harm behaviours, and suicidal behaviours in co-occurrence with cigarette use and marijuana use. |
| Limitations | 19 | Discuss limitations of the study, taking into account sources of potential bias or imprecision. Discuss both direction and magnitude of any potential bias | 24 | This study should be interpreted with consideration of strengths and limitations. Strengths included our use of a large, population-level, nationally representative cohort study; use of sample weighting and, where applicable, adjustment for geographical postcode-level clustering; separation of trans young people from sexuality diverse participants; inclusion of sexuality diverse people identified through sexual attraction and sexual behaviour items in addition to the sexual identity item; and systematic, rigorous approach to quantifying disparities in co-occurring mental ill-health and substance use among LGBTQ+ young people. On the other hand, the small number of trans participants is a limitation of the present study, and results should be interpreted cautiously. The proportion of trans participants in our overall sample, however, is congruent with previous research estimating the number of trans people in Australia and worldwide (between 0.5%-2.7%).^44, 45^ Furthermore, the demographic characteristics of trans and cisgender participants, as shown in Table 1, are relatively comparable. The cross-sectional nature of the present study is another limitation in that longitudinal trend analysis is required to identify the true burden of mental ill-health, substance use, and their co-occurrence among LGBTQ+ young people. |
| Interpretation | 20 | Give a cautious overall interpretation of results considering objectives, limitations, multiplicity of analyses, results from similar studies, and other relevant evidence | 21-24 | We found that LGBTQ+ youth experience significantly more significantly higher levels of psychological distress and higher rates of self-harm and suicidal thoughts/behaviours than their cisgender, heterosexual, and non-sexuality diverse peers, consistent with current literature.^1, 2, 43^ A key finding of the present study was that self-harm behaviour and suicidal thoughts/behaviour disparities were most pronounced among trans young people. Trans young people were 4.9 times as likely than their cisgender peers to engage in self-harm behaviours, and 4.8 times as likely to engage in suicidal thoughts/behaviours. These estimates are lower than in previous research among trans young people in Australia, which may be accounted for by our use of population-representative sampling and inclusion of cisgender comparators.^1, 2^ Moreover, our operationalisation of self-harm and suicidal behaviours rather than strictly ‘attempts’ is important because these other behaviours (e.g., ideation) are significant predictors of future self-harm and suicide attempts among young people and hence represent important targets for prevention and treatment.^46^ Though the magnitudes of these odds ratios are particularly noteworthy, it is important to note the small sample size of trans people albeit representative^47^ may bias these effect estimates.^48^ These findings are a valuable advancement of the literature, given that research on the mental health of LGBTQ+ young people often focuses exclusively on sexuality diverse young people.^49^ Future policy and programs aimed at promoting the mental health of LGBTQ+ young people should prioritise resource allocation toward trans young people and address their unmet, distinct needs given their disproportionate burden of mental ill-health.  This study found that sexuality diverse young people are significantly more likely than their non-sexuality diverse peers to report recent cigarette use and recent marijuana use, with these disparities higher among sexuality diverse females relative to non-sexuality diverse females. Though difficult to compare due to differing timepoints assessed, our findings that over two in five (43%) of sexuality diverse young people report using cigarettes in the preceding 12 months is more than double the one in five (20%) of LGBTQ+ young people reporting ever having used a cigarette in the Writing Themselves in 4 study. Conversely, though discordant measures, our study found that nearly two in five (36%) sexuality diverse young people reported past 12-month marijuana use which is considerably higher than Writing Themselves In 4’s finding that roughly one in three (28%) LGBTQ+ young people reported past six-month marijuana use.^1^ It is important to note that recent substance use does not equate to substance use-related harms nor frequency and intensity of substance use. Hence, these findings of disparities in past 12-month cigarette use and past 12-month marijuana use warrant further attention into the embodied contexts of substance use among LGBTQ+ young people. Such research should consider the positive, altruistic, and social aspects of substance use among LGBTQ+ young people^18, 19^ with the view to advancing LGBTQ+ affirming models of substance use harm reduction. Future longitudinal research is also required, utilising additional population-level, nationally representative datasets to elucidate disparities in substance use among LGBTQ+ young people specifically related to the age of initiation, frequency, and intensity of use through adolescence.  There is a dearth of epidemiological evidence regarding the burden and magnitude of co-occurring mental ill-health and substance use among LGBTQ+ young people. Addressing this research gap, our study found that LGBTQ+ youth in our sample experienced significantly higher rates of co-occurring mental ill-health and substance use than their cisgender, heterosexual, and non-sexuality diverse peers. Specifically, sexuality diverse young people were found to experience significantly higher rates of psychological distress, self-harm behaviours, and suicidal behaviours in co-occurrence with cigarette and marijuana use. While this result converges with and extends previous research finding that sexuality diverse young people are at greater odds of co-occurring depressive symptoms and substance use disorders,^28^ results from many previous studies are largely incomparable insofar as they have utilised samples spanning younger adolescents and adults, or examined mental ill-health and substance use separately.^25, 28, 50^ Researchers and prevention practitioners alike would do well to consider harnessing ‘combined’ models of prevention for co-occurring mental ill-health and substance use^51^ among LGBTQ+ young people, an approach to prevention with demonstrated benefits for concurrently preventing mental ill-health and substance use and related harms during adolescence through early adulthood.^52^ Adapting and evaluating prevention efforts to target co-occurring mental ill-health and substance use is critical for addressing the disproportionate burden, morbidity and mortality of these public health issues among LGBTQ+ young people.^53^  Conversely, our analyses indicated that trans young people were at increased odds of experiencing psychological distress, self-harm behaviours, and suicidal behaviours in co-occurrence with cigarette use and marijuana use. While there is a paucity of research investigating moderating factors between gender diversity and substance use^54^, research suggests trans young people often receive less family acceptance and social support compared with their sexuality diverse peers.^55^ Hence our findings may be explained by previous research suggesting substance use among gender diverse young people may be a coping strategy in response to unique gender minority stressors.^56^ These stressors, including heterosexist stigma and discrimination, are prevalent^5, 49^ and bear deleterious mental ill-health effects for trans young people.^8^ Future research should investigate the interconnected relationship between gender minority stressors and co-occurring mental ill-health and substance among trans young people and investigate modifiable risk and protective factors suitable for prevention and early intervention targeting.  Our observation of higher rates of mental ill-health outcomes among sexuality diverse young females relative to sexuality diverse young males aligns with current literature finding that young females experience higher rates of mental ill-health relative to young men.^29^ Further community-engaged public health research and activities must investigate sexuality diverse young females and non-binary people’s experiences of mental ill-health and assess and address current unmet needs. |
| Generalisability | 21 | Discuss the generalisability (external validity) of the study results | 21-24 | Though the magnitudes of these odds ratios are particularly noteworthy, it is important to note the small sample size of trans people albeit representative^47^ may bias these effect estimates.^48^  Specifically, sexuality diverse young people were found to experience significantly higher rates of psychological distress, self-harm behaviours, and suicidal behaviours in co-occurrence with cigarette and marijuana use. While this result converges with and extends previous research finding that sexuality diverse young people are at greater odds of co-occurring depressive symptoms and substance use disorders,^28^ results from many previous studies are largely incomparable insofar as they have utilised samples spanning younger adolescents and adults, or examined mental ill-health and substance use separately.^25, 28, 50^  On the other hand, the small number of trans participants is a limitation of the present study, and results should be interpreted cautiously. The proportion of trans participants in our overall sample, however, is congruent with previous research estimating the number of trans people in Australia and worldwide (between 0.5%-2.7%).^44, 45^ Furthermore, the demographic characteristics of trans and cisgender participants, as shown in Table 1, are relatively comparable. |
| Other information | |  | | |
| Funding | 22 | Give the source of funding and the role of the funders for the present study and, if applicable, for the original study on which the present article is based | 2 | There are no financial or non-financial interests that are directly or indirectly related to the work submitted for publication. |

*Give information separately for cases and controls in case-control studies and, if applicable, for exposed and unexposed groups in cohort and cross-sectional studies.

**Note:** An Explanation and Elaboration article discusses each checklist item and gives methodological background and published examples of transparent reporting. The STROBE checklist is best used in conjunction with this article (freely available on the Web sites of PLoS Medicine at http://www.plosmedicine.org/, Annals of Internal Medicine at http://www.annals.org/, and Epidemiology at http://www.epidem.com/). Information on the STROBE Initiative is available at www.strobe-statement.org.

References

1. Hill AO, Lyons A, Jones J, McGowan I, Carman M, Parsons M, et al. Writing themselves in 4: The health and wellbeing of LGBTQA+ young people in Australia. Melbourne: Australian Research Centre in Sex, Health and Society, La Trobe University; 2021.

2. Strauss P, Cook A, Winter S, Watson V, Wright Toussaint D, Lin A. Trans Pathways: the mental health experiences and care pathways of trans young people: summary of results. Perth, Australia: Telethon Kids Institute; 2017.

3. Lin Y, Xie H, Huang Z, Zhang Q, Wilson A, Hou J, et al. The mental health of transgender and gender non-conforming people in China: a systematic review. The Lancet Public Health. 2021;6(12):e954-e69.

4. Amos R, Manalastas EJ, White R, Bos H, Patalay P. Mental health, social adversity, and health-related outcomes in sexual minority adolescents: a contemporary national cohort study. The Lancet Child & Adolescent Health. 2020;4(1):36-45.

5. Meyer IH, Russell ST, Hammack PL, Frost DM, Wilson BDM. Minority stress, distress, and suicide attempts in three cohorts of sexual minority adults: A U.S. probability sample. PLOS ONE. 2021;16(3):e0246827.

6. Strauss P, Cook A, Winter S, Watson V, Wright Toussaint D, Lin A. Associations between negative life experiences and the mental health of trans and gender diverse young people in Australia: findings from Trans Pathways. Psychol Med. 2020;50(5):808-17.

7. Tan KKH, Ellis SJ, Schmidt JM, Byrne JL, Veale JF. Mental Health Inequities among Transgender People in Aotearoa New Zealand: Findings from the Counting Ourselves Survey. International Journal of Environmental Research and Public Health [Internet]. 2020; 17(8).

8. Scheer JR, Edwards KM, Sheinfil AZ, Dalton MR, Firkey MK, Watson RJ. Interpersonal Victimization, Substance Use, and Mental Health Among Sexual and Gender Minority Youth: The Role of Self-concept Factors. J Interpers Violence. 2022;37(19-20):Np18104-np29.

9. Day JK, Fish JN, Perez-Brumer A, Hatzenbuehler ML, Russell ST. Transgender Youth Substance Use Disparities: Results From a Population-Based Sample. J Adolesc Health. 2017;61(6):729-35.

10. Watson RJ, Lewis NM, Fish JN, Goodenow C. Sexual minority youth continue to smoke cigarettes earlier and more often than heterosexuals: Findings from population-based data. Drug Alcohol Depend. 2018;184:64-70.

11. Krueger EA, Fish JN, Upchurch DM. Sexual Orientation Disparities in Substance Use: Investigating Social Stress Mechanisms in a National Sample. American Journal of Preventive Medicine. 2020;58(1):59-68.

12. Fish JN, Watson RJ, Porta CM, Russell ST, Saewyc EM. Are alcohol-related disparities between sexual minority and heterosexual youth decreasing? Addiction. 2017;112(11):1931-41.

13. Gamarel KE, Watson RJ, Mouzoon R, Wheldon CW, Fish JN, Fleischer NL. Family Rejection and Cigarette Smoking Among Sexual and Gender Minority Adolescents in the USA. International Journal of Behavioral Medicine. 2020;27(2):179-87.

14. Watson RJ, Fish JN, McKay T, Allen SH, Eaton L, Puhl RM. Substance Use Among a National Sample of Sexual and Gender Minority Adolescents: Intersections of Sex Assigned at Birth and Gender Identity. LGBT Health. 2020;7(1):37-46.

15. Mewton L, Teesson M, Slade T, Grove R. The Epidemiology of DSM-IV Alcohol Use Disorders amongst Young Adults in the Australian Population. Alcohol and Alcoholism. 2011;46(2):185-91.

16. Baker STE, Deady M, Birrell L, Ross K, Fitzpatrick S, Newton N, et al. Prevention of mental and substance use disorders: Shaping priorities for research and implementation. Mental Health & Prevention. 2021;24:200211.

17. Keyes KM, Gary D, O'Malley PM, Hamilton A, Schulenberg J. Recent increases in depressive symptoms among US adolescents: trends from 1991 to 2018. Soc Psychiatry Psychiatr Epidemiol. 2019;54(8):987-96.

18. Freestone J, Bourne A, Layard E, Prestage G, Murray J, Siefried KJ. Playing at the edges, navigating sexual boundaries, and narrating sexual distress; Practices and perspectives of sexuality and gender diverse people who use GHB. Int J Drug Policy. 2022;108:103811.

19. Freestone J, Prestage G, Bourne A, Ezard N, Race K, Nedanoski A, et al. Controlling for pleasure and risk: The experiences of sexuality and gender diverse people who use GHB. Int J Drug Policy. 2022;105:103747.

20. Mewton L, Shaw B, Slade T, Birrell L, Newton NC, Chapman C, et al. The comorbidity between alcohol use and internalising psychopathology in early adolescence. Mental Health & Prevention. 2020;17:200176.

21. Stockings E, Hall WD, Lynskey M, Morley KI, Reavley N, Strang J, et al. Prevention, early intervention, harm reduction, and treatment of substance use in young people. Lancet Psychiatry. 2016;3(3):280-96.

22. Skinner SR, Marino J, Rosenthal SL, Cannon J, Doherty DA, Hickey M. Prospective cohort study of childhood behaviour problems and adolescent sexual risk-taking: gender matters. Sexual Health. 2017;14(6):492-501.

23. Akré ER, Anderson A, Stojanovski K, Chung KW, VanKim NA, Chae DH. Depression, Anxiety, and Alcohol Use Among LGBTQ+ People During the COVID-19 Pandemic. Am J Public Health. 2021;111(9):1610-9.

24. Bränström R, Pachankis JE. Sexual orientation disparities in the co-occurrence of substance use and psychological distress: a national population-based study (2008-2015). Soc Psychiatry Psychiatr Epidemiol. 2018;53(4):403-12.

25. Han BH, Duncan DT, Arcila-Mesa M, Palamar JJ. Co-occurring mental illness, drug use, and medical multimorbidity among lesbian, gay, and bisexual middle-aged and older adults in the United States: a nationally representative study. BMC Public Health. 2020;20(1):1123.

26. Lee JH, Gamarel KE, Kahler CW, Marshall BD, van den Berg JJ, Bryant K, et al. Co-occurring psychiatric and drug use disorders among sexual minority men with lifetime alcohol use disorders. Drug Alcohol Depend. 2015;151:167-72.

27. Mereish EH, Lee JH, Gamarel KE, Zaller ND, Operario D. Sexual orientation disparities in psychiatric and drug use disorders among a nationally representative sample of women with alcohol use disorders. Addict Behav. 2015;47:80-5.

28. Felner JK, Haley SJ, Jun H-J, Wisdom JP, Katuska L, Corliss HL. Sexual orientation and gender identity disparities in co-occurring depressive symptoms and probable substance use disorders in a national cohort of young adults. Addictive Behaviors. 2021;117:106817.

29. Solmi M, Radua J, Olivola M, Croce E, Soardo L, Salazar de Pablo G, et al. Age at onset of mental disorders worldwide: large-scale meta-analysis of 192 epidemiological studies. Molecular Psychiatry. 2022;27(1):281-95.

30. Caspi A, Houts RM, Ambler A, Danese A, Elliott ML, Hariri A, et al. Longitudinal Assessment of Mental Health Disorders and Comorbidities Across 4 Decades Among Participants in the Dunedin Birth Cohort Study. JAMA Network Open. 2020;3(4):e203221-e.

31. Bretherton I, Thrower E, Zwickl S, Wong A, Chetcuti D, Grossmann M, et al. The Health and Well-Being of Transgender Australians: A National Community Survey. LGBT Health. 2020;8(1):42-9.

32. Tan KKH, Wilson AB, Flett JAM, Stevenson Brendan S, Veale JF. Mental health of people of diverse genders and sexualities in Aotearoa/New Zealand: Findings from the New Zealand Mental Health Monitor. Health Promotion Journal of Australia. 2022;33(3):580-9.

33. Chai Y, Luo H, Wei Y, Chan SKW, Man KKC, Yip PSF, et al. Risk of self-harm or suicide associated with specific drug use disorders, 2004–2016: a population-based cohort study. Addiction. 2022;117(7):1940-9.

34. Robinson KH, Bansel P, Denson N, et al. Growing up queer: issues facing young Australians who are gender variant and sexuality diverse: Young and Well Cooperative Research Centre; 2014.

35. Hyde Z, Doherty M, Tilley PJM, McCaul K, Rooney R, Jancey J. The First Australian National Trans Mental Health Study: Summary of Results2015.

36. Elizabeth S, Tiffany J, Roz W, et al. From blues to rainbows: the mental health and well-being of gender diverse and transgender young people in Australia. 2014.

37. Department of Social Services. Growing Up in Australia: The Longitudinal Study of Australian Children (LSAC) - Overview 2022 [Available from: <https://www.dss.gov.au/about-the-department/longitudinal-studies/growing-up-in-australia-lsac-longitudinal-study-of-australian-children-overview#:~:text=LSAC%20commenced%20in%202004%20with,take%20part%20in%20the%20study>.

38. Soloff C, Lawrence D, Johnstone R. LSAC Technical Paper No. 1 - Sample design Canberra, Australia: Australian Institute for Family Studies; 2005.

39. Kessler RC, Andrews G, Colpe LJ, Hiripi E, Mroczek DK, Normand SLT, et al. Short screening scales to monitor population prevalences and trends in non-specific psychological distress. Psychological Medicine. 2002;32(6):959-76.

40. Smout MF. The factor structure and predictive validity of the Kessler Psychological Distress Scale (K10) in children and adolescents. Australian Psychologist. 2019;54(2):102-13.

41. Andrews G, Slade T. Interpreting scores on the Kessler Psychological Distress Scale (K10). Aust N Z J Public Health. 2001;25(6):494-7.

42. Hill AO, Amos N, Lyons A, Jones J, McGowan I, Carman M, Bourne A. Illicit drug use among lesbian, gay, bisexual, pansexual, trans and gender diverse, queer and asexual young people in Australia: Intersections and associated outcomes. Drug and Alcohol Review. 2023;42(3):714-28.

43. Gilbey D, Mahfouda S, Ohan J, Lin A, Perry Y. Trajectories of Mental Health Difficulties in Young People Who are Attracted to the Same Gender: A Systematic Review. Adolescent Research Review. 2020;5(3):281-93.

44. Fisher C, Waling A, Kerr L, Bellamy R, Ezer P, Mikołajczak G, et al. 6th National Survey of Australian Secondary Students and Sexual Health 20182019.

45. Winter S, Diamond M, Green J, Karasic D, Reed T, Whittle S, Wylie K. Transgender people: health at the margins of society. Lancet. 2016;388(10042):390-400.

46. Harris IM, Beese S, Moore D. Predicting future self-harm or suicide in adolescents: a systematic review of risk assessment scales/tools. BMJ Open. 2019;9(9):e029311.

47. Zhang Q, Goodman M, Adams N, Corneil T, Hashemi L, Kreukels B, et al. Epidemiological considerations in transgender health: A systematic review with focus on higher quality data. Int J Transgend Health. 2020;21(2):125-37.

48. Nemes S, Jonasson JM, Genell A, Steineck G. Bias in odds ratios by logistic regression modelling and sample size. BMC Medical Research Methodology. 2009;9(1):56.

49. Russell ST, Fish JN. Mental Health in Lesbian, Gay, Bisexual, and Transgender (LGBT) Youth. Annu Rev Clin Psychol. 2016;12:465-87.

50. Chakraborty P, Alalwan M, Johnson RM, Li L, Lancaster KE, Zhu M. Mental health and substance use by sexual minority status in high school students who experienced sexual violence. Ann Epidemiol. 2021;64:127-31.

51. Teesson M, Newton NC, Slade T, Chapman C, Birrell L, Mewton L, et al. Combined prevention for substance use, depression, and anxiety in adolescence: a cluster-randomised controlled trial of a digital online intervention. The Lancet Digital Health. 2020;2(2):e74-e84.

52. Slade T, Newton NC, Mather M, Barrett EL, Champion KE, Stapinski L, et al. The long-term effectiveness of universal, selective and combined prevention for alcohol use during adolescence: 36-month outcomes from a cluster randomized controlled trial. Addiction. 2021;116(3):514-24.

53. Rees S, Watkins A, Keauffling J, John A. Incidence, Mortality and Survival in Young People with Co-Occurring Mental Disorders and Substance Use: A Retrospective Linked Routine Data Study in Wales. Clin Epidemiol. 2022;14:21-38.

54. Goldbach JT, Tanner-Smith EE, Bagwell M, Dunlap S. Minority Stress and Substance Use in Sexual Minority Adolescents: A Meta-analysis. Prevention Science. 2014;15(3):350-63.

55. Ryan C, Russell ST, Huebner D, Diaz R, Sanchez J. Family acceptance in adolescence and the health of LGBT young adults. J Child Adolesc Psychiatr Nurs. 2010;23(4):205-13.

56. Katz-Wise SL, Sarda V, Austin SB, Harris SK. Longitudinal effects of gender minority stressors on substance use and related risk and protective factors among gender minority adolescents. PLOS ONE. 2021;16(6):e0250500.
