# Supplementary material for: Prevalence, distribution, and inequitable co-occurrence of mental ill-health and substance use among gender and sexuality diverse young people in Australia: epidemiological findings from a population-based cohort study
Source: Soc Psychiatry Psychiatr Epidemiol. 2024 Jul 23;59(12):2323–37. doi: 10.1007/s00127-024-02714-1 (PMC11522107; doi:10.1007/s00127-024-02714-1)
Supplement: Supplementary file 2 — Supplementary Material 2 [file 127_2024_2714_MOESM2_ESM.docx]

**Table 1. Prevalence and distribution of psychological distress, self-harm, and suicidality among LGBTQ+ young people in The Longitudinal Study of Australian Children**

|  | Trans vs Cis | | | Heterosexual vs Gay/Lesbian / Bisexual / Other | | | | | | | Non-Sexuality diverse vs Sexuality Diverse | | | Female Non-Sexuality Diverse vs Female Sexuality Diverse | | | Male Non-Sexuality Diverse vs Male Sexuality Diverse | | |
| --- | --- | --- | --- | --- | --- | --- | --- | --- | --- | --- | --- | --- | --- | --- | --- | --- | --- | --- | --- |
|  | Cis (ref.) | Trans |  | Heterosexual (ref.) | Gay/Lesbian |  | Bisexual |  | Other |  | Non-Sexuality Diverse (ref.) | Sexuality Diverse |  | Female Non-Sexuality Diverse (ref.) | Female Sexuality Diverse |  | Male Non-Sexuality Diverse (ref.) | Male Sexuality Diverse |  |
|  | N  % | N  % | aOR  95% CI  *p* | N  % | N  % | aOR  95% CI  *p* | N  % | aOR  95% CI  *p* | N  % | aOR  95% CI  *p* | N  % | N  % | aOR  95% CI  *p* | N  % | N  % | aOR  95% CI  *p* | N  % | N  % | aOR  95% CI  *p* |
| High or very high levels of psychological distress | 871  33.4 | 25  63.6 | 3.5  1.49-8.11  0.004 | 648  29.0 | 42  68.3 | 5.3  2.64-10.56  *<0.001* | 155  64.8 | 4.5 3.29-6.20  *<0.001* | 19  59.3 | 3.6 1.71- 7.44  *<0.001* | 623  28.6 | 248  58.7 | 3.5 2.73 – 4.60  <0.001 | 364  36.6 | 157  61.3 | 2.7  1.98 – 3.82  *<0.001* | 258  21.9 | 73  49.8 | 3.5  2.29 – 5.51  *<0.001* |
| Any past 12-month self-harm thoughts/behaviour | 382  14.7 | 18  45.5 | 4.9  2.21-10.68  <0.001 | 262  11.7 | 44  28.4 | 3.0  1.53-5.85  *0.001* | 91  38.5 | 4.7 3.24-6.83  *<0.001* | 14  44.0 | 5.9 2.95 – 11.78 *<0.001* | 247  11.4 | 139  33.3 | 3.9  2.89 – 5.23  <0.001 | 132  13.3 | 88  34.8 | 3.5 2.35 – 5.16 *<0.001* | 113  9.6 | 39  26.5 | 3.4 2.03-5.68 *<0.001* |
| Past 12 months self-harm thoughts | 307  11.8 | 14  35.2 | 4.1  1.85-8.94  <0.001 | 210  9.4 | 16  26.2 | 3.4  1.66-7.02  *<0.001* | 72  30.2 | 4.2 2.78 – 6.25 *<0.001* | 13  41.6 | 6.8 3.40-13.81  *<0.001* | 197  9.0 | 117  27.8 | 3.9  2.80-5.33  <0.001 | 101  10.1 | 76  29.8 | 3.8 2.47-5.75 *<0.001* | 94  7.9 | 32  22.1 | 3.3 1.92-5.62 *<0.001* |
| Past 12 months self-harm behaviour | 234  9.0 | 14  35.4 | 5.5  2.48-12.38  <0.001 | 159  7.1 | 14  23.5 | 4.0  1.90-8.43  *<0.001* | 55  23.3 | 4.0 2.59-6.04 *<0.001* | * | 4.3 1.92-9.66 *<0.001* | 146  6.7 | 90  21.5 | 3.8 2.69-5.38 <0.001 | 80  8.1 | 54  21.3 | 3.1 1.93-4.87 *<0.001* | 65  5.5 | 26  17.4 | 3.7 1.94-6.88 *<0.001* |
| Any past 12-month suicidal thoughts/behaviour | 396  15.2 | 18  46.3 | 4.8  2.26-10.17  <0.001 | 280  12.5 | 18  29.1 | 2.9  1.44-5.70  *<0.001* | 91  38.3 | 4.3 2.99 – 6.28 *<0.001* | 14  44.3 | 5.5 2.80 – 10.99 *<0.001* | 267  12.2 | 140  33.2 | 3.6 2.66 – 4.80 <0.001 | 130  13.1 | 89  35.1 | 3.6 2.46 – 5.27  *<0.001* | 134  11.4 | 39  26.3 | 2.8  1.67 – 4.63  *<0.001* |
| Past 12 months suicidal thoughts | 307  11.8 | 14  35.2 | 4.1  1.85-8.94  *<0.001* | 210  9.4 | 16  26.2 | 3.4  1.66-7.02  *<0.001* | 72  30.2 | 4.2 2.78-6.25 *<0.001* | 13  41.6 | 6.8 3.40-13.81 *<0.001* | 197  9.0 | 117  27.8 | 3.9 2.80-5.33 *<0.001* | 101  10.1 | 76  29.8 | 3.8  2.47-5.72 *<0.001* | 94  7.9 | 32  22.1 | 3.3 1.92-5.62 *<0.001* |
| Past 12 months suicidal planning | 234  9.0 | 13  34.1 | 5.2  2.43-11.30  *<0.001* | 168  7.5 | 12  20.2 | 3.1 1.36-7.08 *0.007* | 50  21.1 | 3.3 2.16-4.99 *<0.001* | * | *3.6 1.51-8.47 0.0037* | 157  7.2 | 84  19.9 | 3.2 2.28-4.45 *<0.001* | 70  7.1 | 48  18.9 | 3.1  1.92-4.86 *<0.001* | 86  7.3 | 26  17.7 | 2.8 1.53-4.93 *<0.001* |
| Past 12 months suicide attempt | 151  5.8 | * | 3.0  1.14-8.02  *0.03* | 103  4.6 | * | 4.1 1.60-10.37 *0.003* | 36  14.9 | 3.6 2.18-6.03 *<0.001* | * | 2.0  0.45-8.87 *0.4* | 94  4.3 | 58  13.6 | 3.5  2.25-5.39 *<0.001* | 49  4.9 | 32  12.7 | 2.8  1.48-5.33 *0.0* | 46  3.8 | 20  13.5 | 3.9  1.93-7.92 *0.0002* |
| Past 12 months alcohol use | 29 78.0 | 2100 (87.2) | 0.5  0.2, 1.5  *0.2* | 1791 (86.8) | 51 (87.5) | 1.1  0.3, 3.4  *0.9* | 212 (93.2) | 2.1  1.1, 4.1  *0.03* | 17 (64.0) | 0.3  0.1,  0.6  *0.03* | 1760 (87.4) | 310 (85.9) | 0.9  0.6, 1.3  *0.5* | 822 (87.5) | 191 (87.7) | 0.9  0.6, 1.3  *0.5* | 937 (87.3) | 101 (82.2) | 0.7  0.4, 1.3  *0.2* |
| Past 12 months cigarette use | 18 (50.8) | 875 (36.9) | 1.8  0.8, 4.1  *0.2* | 733  (36.1) | 28 (48.9) | 1.7  0.9, 3.1  *0.09* | 104 (46.5) | 1.5  1.1, 2.2  *0.02* | * | 0.5  0.2, 1.1  *0.07* | 715 (36.2) | 156 (43.1) | 1.3  1.0, 1.8  *0.04* | 298 (32.6) | 92 (42.9) | 1.6  1.1, 2.2  *0.01* | 416 (39.1) | 54 (42.3) | 1.1  0.7, 1.8  *0.6* |
| Past 12 months marijuana use | * | 706 (22.9) | 0.70.3, 1.8  *0.5* | 580 (28.3) | 20 (36.0) | 1.4  0.8, 2.7  *0.3* | 96 (42.4) | 1.9  1.3, 2.6  *<0.001* | * | 0.3  0.1, 1.0  *0.05* | 568 (28.4) | 132 (36.4) | 1.4  1.1, 1.9  *0.01* | 219 (23.6) | 79 (36.2) | 1.8  1.3, 2.6  *<0.001* | 348 (32.5) | 46 (36.9) | 1.2  0.8, 2.0  *0.4* |

* Cell counts <10 are censored to prevent re-identification of participants as required by the Australian Institute of Family Studies.

**Table 2. Multinominal logistic regression models estimating associations between sexuality diversity with outcomes of co-occurring mental ill-health and substance use among sexuality diverse young people**

|  | Sexual orientation |  |  |
| --- | --- | --- | --- |
|  | Non-Sexuality Diverse | Sexuality Diverse |  |
|  | MOR^[[1]](#footnote-2)^ | MOR (95% CI^[[2]](#footnote-3)^) | *p-value* |
| Outcome 1: High or very high levels of psychological distress (K10^[[3]](#footnote-4)^) and Recent Cigarette Use (CU^[[4]](#footnote-5)^) |  |  |  |
| No K10, No CU | 1.0 | - | - |
| K10 Only | 1.0 | 5.2 (2.31-11.51) | <0.001 |
| CU Only | 1.0 | 1.5 (0.75-3.06) | <0.001 |
| K10 and CU | 1.0 | 6.7 (3.39-13.19) | <0.001 |
| Outcome 2: High or very high levels of psychological distress (K10) and Recent Alcohol Use (AU^[[5]](#footnote-6)^) |  |  |  |
| No K10, No AU | 1.0 | - | - |
| K10 Only | 1.0 | 1.0 (0.35-1.20) | <0.001 |
| AU Only | 1.0 | 0.4 (0.17-1.02) | 0.250 |
| K10 and AU | 1.0 | 1.7 (0.70-4.26) | <0.001 |
| Outcome 3: High or very high levels of psychological distress (K10) and Recent Marijuana Use (MU^[[6]](#footnote-7)^) |  |  |  |
| No K10, No MU | 1.0 | - | - |
| K10 Only | 1.0 | 1.1 (0.66-2.00) | <0.001 |
| MU Only | 1.0 | 0.7 (0.44-1.25) | <0.001 |
| K10 and MU | 1.0 | 3.1 (1.89-4.94) | <0.001 |
| Outcome 4: Recent Self-harm Behaviours (SH^[[7]](#footnote-8)^) and Recent Cigarette Use (CU) |  |  |  |
| No SH, No CU | 1.0 | - | - |
| SH Only | 1.0 | 7.0 (2.92-16.64) | <0.001 |
| CU Only | 1.0 | 1.4 (0.83-2.31) | 0.250 |
| SH and CU | 1.0 | 6.9 (3.97-12.04) | <0.001 |
| Outcome 5: Recent Self-harm Behaviours (SH) and Recent Alcohol Use (AU) |  |  |  |
| No SH, No AU | 1.0 | - | - |
| SH Only | 1.0 | 0.0 | <0.001 |
| AU Only | 1.0 | 0.4 (0.20-0.86) | 0.250 |
| SH and AU | 1.0 | 1.9 (0.90-4.02) | <0.001 |
| Outcome 6: Recent Self-Harm Behaviours (SH) and Recent Marijuana Use (MU) |  |  |  |
| No SH, No MU | 1.0 | - | - |
| SH Only | 1.0 | 1.0 (0.56-1.69) | <0.001 |
| MU Only | 1.0 | 0.9 (0.58-1.42) | 0.250 |
| SH and MU | 1.0 | 4.3 (2.61-7.10) | <0.001 |
| Outcome 7: Recent Suicidal Behaviour (SB^[[8]](#footnote-9)^) and Recent Cigarette Use (CU) |  |  |  |
| No SB, No CU | 1.0 | - | - |
| SB Only | 1.0 | 5.6 (2.28-13.93) | <0.001 |
| CU Only | 1.0 | 1.3 (0.80-2.15) | 0.250 |
| SB and CU | 1.0 | 5.9 (3.41-10.04) | <0.001 |
| Outcome 8: Recent Suicidal Behaviour (SB) and Recent Alcohol Use (AU) |  |  |  |
| No SB, No AU | 1.0 | - | - |
| SB Only | 1.0 | 0.0 | - |
| AU Only | 1.0 | 0.4 (0.20-0.86) | 0.25 |
| SB and AU | 1.0 | 1.7 (0.81-3.63) | <0.001 |
|  |  |  |  |
| Outcome 9: Recent Suicidal Behaviour (SB) and Recent Marijuana Use (MU) |  |  |  |
| No SB, No MU | 1.0 | - | - |
| SB Only | 1.0 | 1.0 (0.68-1.03) | <0.001 |
| MU Only | 1.0 | 1.0 (0.65-1.58) | 0.250 |
| SB and MU | 1.0 | 3.8 (2.27-6.27) | <0.001 |

**Table 3. Associations between young people’s gender identities and co-occurring mental ill-health and substance use outcomes**

|  | Gender identity | |  |
| --- | --- | --- | --- |
|  | Cisgender | Trans |  |
|  | N | N | *p-value* |
| Outcome 1: High or very high levels of psychological distress (K10) and Recent Cigarette Use (CU) | 345 | 11 | 0.020 |
| Outcome 2: High or very high levels of psychological distress (K10) and Recent Alcohol Use (AU) | 701 | 20 | 1 |
| Outcome 3: High or very high levels of psychological distress (K10) and Recent Marijuana Use (MU) | 288 | $8$ | 0.21 |
| Outcome 4: Recent Self-harm Behaviours (SH) and Recent Cigarette Use (CU) | 176 | 9 | <0.001 |
| Outcome 5: Recent Self-harm Behaviours (SH) and Recent Alcohol Use (AU) | 300 | 15 | 1 |
| Outcome 6: Recent Self-Harm Behaviours (SH) and Recent Marijuana Use (MU) | 154 | 6 | 0.017 |
| Outcome 7: Recent Suicidal Behaviour (SB) and Recent Cigarette Use (CU) | 180 | 8 | 0.001 |
| Outcome 8: Recent Suicidal Behaviour (SB) and Recent Alcohol Use (AU) | 304 | 14 | 1 |
| Outcome 9: Recent Suicidal Behaviour (SB) and Recent Marijuana Use (MU) | 150 | 4 | 0.061 |

*In accordance with Longitudinal Study of Australian Children dataset governance rules, the authors have censored cell counts <10.

1. Multinomial odds ratio [↑](#footnote-ref-2)
2. 95% confidence interval [↑](#footnote-ref-3)
3. High or very high levels of psychological distress as self-reported on the Kessler Psychological Distress 10-item Scale [↑](#footnote-ref-4)
4. Past 12-month cigarette use [↑](#footnote-ref-5)
5. Past 12-month alcohol use [↑](#footnote-ref-6)
6. Past 12-month marijuana use [↑](#footnote-ref-7)
7. Past 12-month self-harm ideation or attempts [↑](#footnote-ref-8)
8. Past 12-month suicidal ideation, planning or attempts [↑](#footnote-ref-9)
